# Design and Control of Supramolecular Structure in Crown Ether–Manganese Thiocyanate Complexes Tuned by Aliphatic Diamine Alkyl Chains: Parity-Dependent Modulation of Dielectric and Electrochemical Properties

**DOI:** 10.3390/molecules31122012

**Published:** 2026-06-09

**Authors:** Tong Zhang, Hongzhi Hu, Adila Abuduheni, Yang Liu, Zunqi Liu

**Affiliations:** 1 Chemistry and Chemical Engineering College, Xinjiang Agricultural University, Urumqi 830052, China; tongzhang070300@163.com (T.Z.); 17799751675@163.com (A.A.); 2 Xinjiang Key Laboratory for High Value Utilization of Agricultural and Livestock By-Products, Urumqi 830052, China; 3 Xinjiang Sub-Center National Engineering Research Center of Novel Equipment for Polymer Processing, Urumqi 830052, China

**Keywords:** alkyl chain of aliphatic diamine, 18-crown-6, dielectric properties, molecular-level design, crystal materials

## Abstract

Aliphatic diamines possess two amino functional groups and exhibit diverse chemical properties and tunable molecular structures. By selecting the guest [(C_2_H_2n+4_N_2_), *n* = 2–6] and host 18-crown-6, and controlling the design and assembly processes via modulation by thiocyanate and a manganese salt, a series of dumbbell-shaped crown ether complexes, (C_2_H_2n+6_N_2_)^2+^(18-crown-6)_2_[Mn(NCS)_4_]^2−^·(δ_n_,_2_C_2_H_3_N), *n* = 2–6, (**1**)–(**5**), was synthesized and analyzed by single-crystal X-ray diffraction (SCXRD) at 100 K and 293 K. Variable-temperature infrared and XRD analyses confirmed that compounds **3** and **5** underwent a phase transition. As the length of the carbon chain increases and alternates between odd and even, the interplanar dihedral angle of the crown ether exhibits a distinct pattern: Even-number chains arrange in parallel, whereas odd-number chains form a pronounced angle. This structural pattern influences macroscopic deformation of the crystal and induces corresponding periodic variations in the dielectric and electrochemical properties. The wide-bandgap insulators and magnetic properties are primarily governed by the inorganic components of the system and are less influenced by the organic portion. This study reveals principles for regulating supramolecular conformation and functional properties through the parity of the organic chain lengths, providing a strategy for the molecular-level design of supramolecular crystal materials with ordered structures and tunable properties.

## 1. Introduction

Aliphatic diamines (C_2_H_2n+4_N_2_) are widely used as flexible bridging ligands in coordination polymers and metal–organic frameworks (MOFs) [1,2,3,4,5,6]. The chain length directly influences the topological structure and supramolecular interactions of these molecules. Short chains (*n* = 2–3: ethylenediamine/1,3-propanediamine) favor the formation of compact structures and induce ligand-field strain. Thus, they enhance the effect of the ligand field on the metal centers. Long chains (*n* = 4–6: butanediamine/pentanediamine/hexanediamine) introduce conformational freedom, promoting interpenetration, van der Waals interactions, and guest inclusion [7,8,9]. However, the systematic role of diamine chain length in regulating the supramolecular conformation and multifunctional properties of crown-ether-based hybrid materials remains underexplored.

Since their discovery by Pedersen in 1967, crown ethers have become a cornerstone of supramolecular chemistry because of their unique cavity structures and cation-recognition capabilities. 18-Crown-6 exhibits exceptional affinity for alkali-metal ions and ammonium cations because the diameter of its cavity (2.6–3.2 Å) is highly compatible with the ionic radii of target ions such as K^+^ (1.33 Å) [10,11,12,13]. In crown ether systems, diamines can serve the dual roles of hydrogen bond donors (via NH_2_ groups), forming 2D layers or 3D networks with the oxygen atoms of the crown ether, and charge-compensating cations (e.g., (C_2_H_2n+6_N_2_)^2+^) that, upon protonation, serve as templates for the arrangement of the crown ether and assembly of the anions [14,15,16]. In particular, 18-crown-6 exhibits high selectivity for ammonium cations, making it an ideal host for encapsulating protonated aliphatic diamines. Recent studies have shown that multidimensional structures with newly recognized functions, such as dielectric switching capability, magnetic bistability, and ion-transport ability, can be generated when organic cation–crown ether composite building blocks are assembled with anionic metal complexes [17,18,19,20,21,22,23,24,25]. Nevertheless, systematic investigations into how the length of the diamine spacer regulates these properties in manganese–thiocyanate–crown ether systems are still lacking.

Manganese (II) complexes exhibit rich coordination chemistry and diverse physical properties derived from their high-spin d^5^ electron configuration. These properties include paramagnetic behavior and high sensitivity to ligand fields [26,27,28,29,30]. The thiocyanate ion (SCN^−^), with dual-donor capability (S or N coordination) and bridging modes (μ-1,3 or μ-1,1,3), serves as a key ligand in magneto-structural chemistry [31,32,33,34,35]. When combined with 18-crown-6 and protonated diamines, these components can form hydrogen-bonded networks that undergo temperature-induced phase transitions accompanied by dielectric anomalies. For example, the compound [(Gly)^2+^(18-crown-6)_2_(MnCl_4_)^2−^] undergoes a temperature-induced structural transition from *P*2_1_/c (100 K) to *C*2/c (293 K), accompanied by dielectric anomalies associated with fluctuations of the O-H···Cl hydrogen bond [36]. These behaviors highlight the potential of manganese–crown ether hybrid systems as switchable materials.

Based on the above considerations, this study aims to systematically investigate the effect of progressively increasing the diamine chain length (*n* = 2–6) on the supramolecular assembly, thermally induced phase transition and multi-physical performances of five new hybrid complexes: (C_2_H_2n+6_N_2_)^2+^(18-crown-6)_2_[Mn(NCS)_4_]^2−^·(δ_n_,_2_C_2_H_3_N), *n* = 2–6, (**1**)–(**5**), We have synthesized these five compounds and characterized them by single-crystal X-ray diffraction (100 K and 293 K), variable-temperature infrared spectroscopy, thermogravimetry, differential scanning calorimetry, powder X-ray diffraction, dielectric measurements, solid-state UV–vis spectroscopy, density of states calculations, Hirshfeld surface analysis, and cyclic voltammetry. Our results reveal a clear even–odd alternation in the dihedral angle of the crown ethers depending on the parity of the diamine carbon chain length: even-numbered chains arrange in parallel, whereas odd-numbered chains form a pronounced angle. This structural pattern directly influences the macroscopic deformation of the crystal and induces corresponding periodic variations in the dielectric and electrochemical properties. In contrast, the semiconductor and magnetic properties are primarily governed by the inorganic [Mn(NCS)_4_]^2−^ units and are less influenced by the organic portion. This study elucidates the principles for regulating supramolecular conformation and functional properties through the parity of organic chain lengths, providing a molecular-level design strategy for supramolecular crystal materials with ordered structures and tunable properties.

## 2. Analysis of the Crystal Structure of Compounds

### 2.1. Crystallography: Evolution of Symmetry and Crystal System Transitions

Figure S1 presents a schematic of the synthesis of compounds **1**–**5**. Detailed synthetic procedures for all compounds are provided in the Supporting Information. Single-crystal XRD data were collected at 100 K (LT) and 293 K (RT), and the measured intensities revealed the structure of compounds **1**–**5**. The CCDC deposition numbers for compounds **1**–**5** at low temperatures are 2534434, 2534436, 2537285, 2537323 and 2537334 at LT, and 2534435, 2534437, 2537286, 2537324 and 2537335 at RT. The experimental results showed high consistency with the theoretical predictions. Figure S2 shows the simplest component diagrams for compounds **1**–**5** at 100 K and 293 K. At 100 K or 293 K, the minimal composition of compounds **1**–**5** comprises one molecule of an inorganic-metal-containing anion in a tetrahedral configuration [Mn(NCS)_4_]^2−^, one molecule of a diprotonated diamine cation (ethylenediamine, propylenediamine, butylenediamine, pentylenediamine, or hexamethylenediamine, respectively), and two molecules of 18-crown-6 ether. At 100 K and 293 K, in addition to the above components, compound **1** also contains one molecule of acetonitrile, and the crown ether lies on a center of symmetry. At low temperatures, one crown ether molecule in compound **2** undergoes disorder, whereas at high temperatures, both crown ether molecules become disordered. Compound **3** exhibits significant changes: at low temperature, the asymmetric unit contains approximately twice the number of components compared to the high-temperature structure. Compound **4** shows no significant structural change. The hexamethylenediamine ions in compound **5** are disordered. Moreover, the bond lengths and angles of the organic cations in compounds **1**–**5** exhibit varying degrees of alteration at both room and low temperatures (Tables S2–S6). This systematic increase in diamine chain length (rather than the observed disorder) readily induces systematic changes in the physical properties of the materials.

As the length of the diamine chain increased, the organic components became more flexible, leading to crystallization in a higher-symmetry space group. The data for compounds **1**–**5** are presented in Table S1. Through systematic investigation of the crystal structures of this series of compounds, we identified a clear crystallographic pattern: as the length of the diamine chain increases, the crystal systems exhibit a systematic transition from low symmetry to high symmetry, with a corresponding improvement in the packing efficiency. Specifically, Compounds **2** and **3** both crystallize in the triclinic crystal system (space group *P*-1), which exhibits the lowest symmetry. As the chain length increases, the low-temperature phases of compounds **4** and **5** transition to the monoclinic crystal system (space groups *P*2_1_/n and *P*2_1_/c, respectively). Compound **5**, with the longest chain length, crystallizes in the orthorhombic system (*P*nma), with higher symmetry at elevated temperatures. This evolutionary path (triclinic → monoclinic → orthorhombic) clearly demonstrates that increasing the length of the molecular chain enhances the regularity of the molecular arrangement and symmetry of the crystal lattice, thereby enabling more efficient packing. The sole exception is compound **1**. Compound **1**, which contains the shortest and most rigid ethylenediamine chain (*n* = 2), does not readily form a stable crystal lattice by itself. Notably, an acetonitrile solvent molecule is present in the asymmetric unit of **1** (Figure S2). This solvent molecule likely fills space within the crystal lattice and participates in hydrogen-bonding interactions (Tables S8–S12), thereby contributing to the overall stability and the observed orthorhombic symmetry (space group *P*nma). The role of solvent molecules in stabilizing crystal structures is well documented; for example, Kai et al. [37] demonstrated that solvent composition can modulate intermolecular interactions and induce polymorphic transitions, and Liwen et al. [38] showed that crystallization solvents participate in hydrogen-bonding networks to reinforce ordered molecular packing. This demonstrates the crucial role of solvent molecules in crystal structures. Detailed crystallographic data for compounds **1**–**5** are summarized in Table S1.

### 2.2. Cationic Structure: Regulation of the Dihedral Angle of Crown Ethers by the Parity of Diamine Chain Lengths

**Figure 1:**
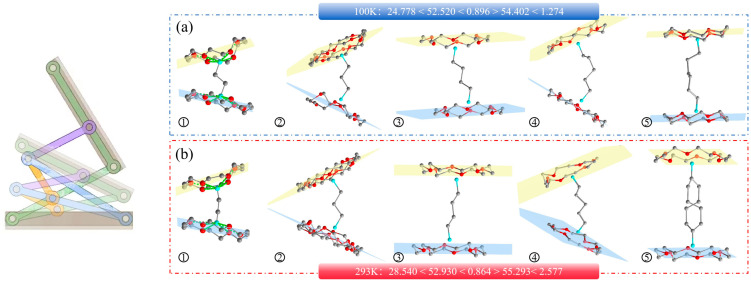
Structural diagrams of the sandwich-type crown ether-diammonium supramolecular dication in compounds **1**–**5** at LT (**a**) and RT (**b**).

**Figure 2:**
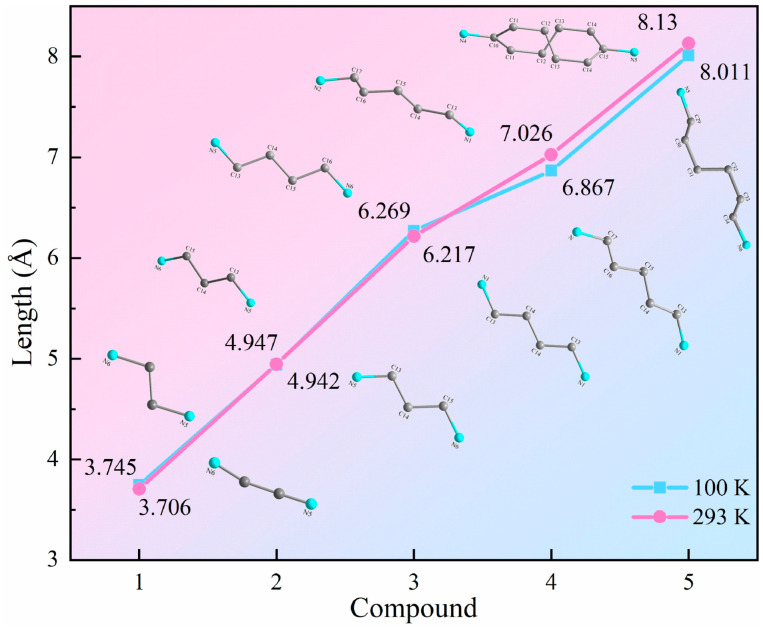
Distance between two N atoms in diamine ions of compounds **1**–**5** at LT and RT.

Analysis of the sandwich-type crown ether–diammonium supramolecular dication structure (Figure 1) shows that the angle between the two crown ethers changes systematically with increasing length of the diamine chain. Similarly, the angle of the diamine chain exhibits regular variations with temperature. This behavior is akin to a telescoping frame, inducing changes in the physical properties of the material. Initially, the angles between the atoms in the diamine chain are small. As the temperature increases, these interatomic angles expand, causing the diamine chain to elongate. Measurements of the distance between the two nitrogen atoms in the diamine ions revealed that compounds **1** and **3** exhibit anomalous behavior, where the diamine chain becomes elongated at low temperatures (Table S7, Figure 2). Structural analysis indicated that at 100 K, the hydrogen bonding interactions between the acetonitrile molecules, crown ether molecules, and diamine molecules in compound **1** are weaker. Consequently, the diamine chain experiences less constraint from hydrogen bonding, leading to elongation of the molecular chain. At 293 K, hydrogen bonding interactions become stronger, causing the diamine chain to shorten accordingly. The diamine chains in compound **3** also exhibit shortening owing to changes in the hydrogen bonding forces. The remaining compounds all exhibit expansion at higher temperatures, leading to an increase in the chain length of the diamine. The hydrogen-bond length and bond angle data are presented in Tables S8–S12.

### 2.3. Hydrogen-Bond Networks and Packing Structures: From Helices to Parallel Arrangements

Compounds **1**–**5** all form one-dimensional, infinitely extended chain-like supramolecular structures through hydrogen bonding, consisting of alternating arrangements of discrete inorganic anions [Mn(NCS)_4_]^2−^ and diprotonated diamine–crown ether cations (Figures S4 and S5). The one-dimensional chains of each compound extend along well-defined crystallographic directions and exhibit distinct extension characteristics at LT and RT: at both low temperatures and room temperature, the chains of Compound **1** extend along the c-axis within the a–c plane, and their orientation remains stable; at both low temperatures and room temperature, the chains of Compound **2** extend along the a-axis within the a–b plane, with no change observed with temperature; for compound **3**, at 100 K it crystallizes in the triclinic system (space group *P*-1), and the chains extend along the b-axis within the a–b plane; at 293 K it crystallizes in the monoclinic system (space group C2/c). Under the conventional axis setting at RT, the apparent extension direction becomes the c-axis within the a–c plane. However, this apparent change does not correspond to a physical reorientation of the structural motif; rather, it arises from the redefinition of crystallographic axes according to standard conventions upon the symmetry change. If a consistent non-standard space-group setting is adopted (e.g., using the B2/b11 setting for the RT structure to maintain axis correspondence with the LT structure), the actual chain direction remains unchanged. Similarly, for compound **5**, at 100 K it crystallizes in the monoclinic system (space group *P*2_1_/c), with chains extending along the c-axis within the b–c plane; at 293 K it crystallizes in the orthorhombic system (space group *P*nma). Using the non-standard *P*bnm setting to preserve axis correspondence, the chain direction does not undergo physical reorientation. At both low temperatures and room temperature, the chains of Compound **4** extend in a zigzag pattern within the b–c plane, parallel to the b-axis, and the structure remains stable. The above results indicate that the one-dimensional chains of compounds **1**, **2**, and **4** remain structurally stable over the temperature range of 100–293 K, with their propagation direction remaining constant regardless of temperature. In contrast, compounds **3** and **5** undergo structural phase transitions from lower to higher symmetry upon heating, which are accompanied by the conventional redefinition of crystallographic axes; this leads to an apparent change in the chain propagation direction, but the actual packing topology of the supramolecular chains within the crystal does not undergo physical reorientation. This is fully consistent with the phase transitions observed in temperature-programmed XRD, DSC, and infrared spectroscopy.

**Figure 3:**
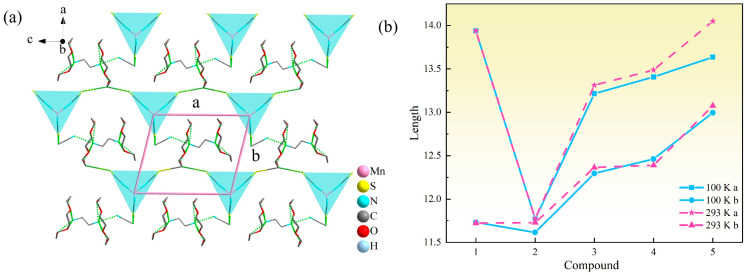
Two-dimensional hydrogen-bonding network of compound **1** at LT: (**a**) side lengths of quadrilaterals constructed with manganese atoms as vertices for compounds **1**–**5**; the longer sides are designated as “a” and the shorter sides as “b”. Data at LT and RT are presented for each compound where applicable (**b**).

Figure 3a displays the hydrogen-bond network structures of compound **1** at 100 K. Under the 100 K and 293 K temperature conditions, four adjacent thiocyanate–manganese complexes lying in the same plane within this space group were selected. Quadrilaterals were constructed with manganese atoms as vertices. The longer sides of these quadrilaterals were designated as “a” and the shorter sides as “b”. Consistent with the expectation of synchronous expansion of the parallelogram framework owing to elongation of the flexible alkyl chains, the length of both sides (a and b) increased proportionally as the chain length of the diamine increased. The length of sides a and b of the quadrilateral increased correspondingly. The long edge (a) of Compound **1** exhibits a distinct anomaly with irregular variations, attributable to the presence of acetonitrile molecules within the unit cell. However, upon heating, the edge length of compound **1** remained virtually unchanged, attributed to the robust anchoring of the chain ends by N-H···O and N-H···N hydrogen bonds. The slight changes in the edge lengths of the remaining compounds result from the synergistic effects of the chain length, hydrogen bonding interactions, and cation orientation, which collectively determine the evolution of the framework dimensions at different temperatures.

The crystal packing structures of compounds **1**–**5** at low temperature (100 K) and room temperature (293 K) are shown in Figure S7. All compounds are assembled from discrete inorganic anions [Mn(NCS)_4_]^2−^ and diprotonated diamine–bis(18-crown-6) supramolecular cations via hydrogen bonding and van der Waals forces. Under low-temperature conditions (Figure S7a–e), the arrangement of organic cations and inorganic anions is highly ordered, molecular thermal motion is significantly suppressed, and the hydrogen-bond network is well-organized. The supramolecular cations of compounds **1**, **2**, and **4** exhibit a certain tilt angle within the layer, forming a helical stacking arrangement; in contrast, the supramolecular cations of compounds **3** and **5** are arranged nearly parallel to one another, exhibiting an in-plane parallel stacking pattern. This pattern is fully consistent with the results showing that the dihedral angle between crown ether rings is regulated by the odd–even effect of the diamine carbon chains. Upon heating to room temperature (Figure S7f–j), molecular thermal motion increased, the disorder of the crown ether and alkyl diamine chains rose, and some hydrogen bonds relaxed. The overall stacking topology of compounds **1**, **2**, and **4** remained stable, with no significant structural rearrangement observed; in contrast, compounds **3** and **5** underwent reversible structural phase transitions, resulting in marked changes in their stacking patterns. Notably, the crown ether moiety in compound **2** exhibited more pronounced dynamic disorder, leading to a decrease in local stacking regularity, which is directly related to its antiferromagnetic behavior. Overall, the stacking patterns of this series of compounds are primarily determined by the odd–even effect of the diamine carbon chain; temperature changes mainly affect the local order of the organic cations and the strength of hydrogen bonds. Compounds **1**, **2**, and **4** maintain a constant stacking topology within the temperature range of 100–293 K, while only compounds **3** and **5** undergo reversible structural phase transitions.

## 3. Results and Discussion

### 3.1. Infrared Spectral Analysis of Compounds ***1***–***5***

The main components of compound **1** were preliminarily determined using infrared (IR) spectroscopy. An appropriate amount of each compound was ground to a powder in a mortar, mixed thoroughly with pure, dried potassium bromide, and pressed into a transparent disk without cracks. This disk was scanned in the range of 4000–500 cm^−1^. The results for all samples are shown in Figure S6. The functional groups corresponding to the absorption bands in the infrared spectrum were assigned as follows: The absorption bands at 3079 and 2912 cm^−1^ correspond to the N-H stretching vibrations of the diamine and -CH_2_- group, respectively. The sharp, intense absorption band at 2062 cm^−1^ and the peak at 480 cm^−1^ correspond to the stretching and bending vibrations of the -N=C=S group in potassium thiocyanate, respectively. The absorption band near 1600 cm^−1^ represents the N-H bending vibration of the amine group. The three strong absorption bands at 1096, 959, and 835 cm^−1^ correspond to the bending vibration of -C-O-C- in 18-crown-6. The peak at 1349 cm^−1^ represents the bending vibration of the methylene -CH_2_- group. Analysis of the infrared spectrum indicates that all four components are present in compounds **1**–**5**. Furthermore, with increasing length of the diamine chain, the bending-vibration peak of the methylene -CH_2_- at 1349 cm^−1^ progressively intensifies.

**Figure 4:**
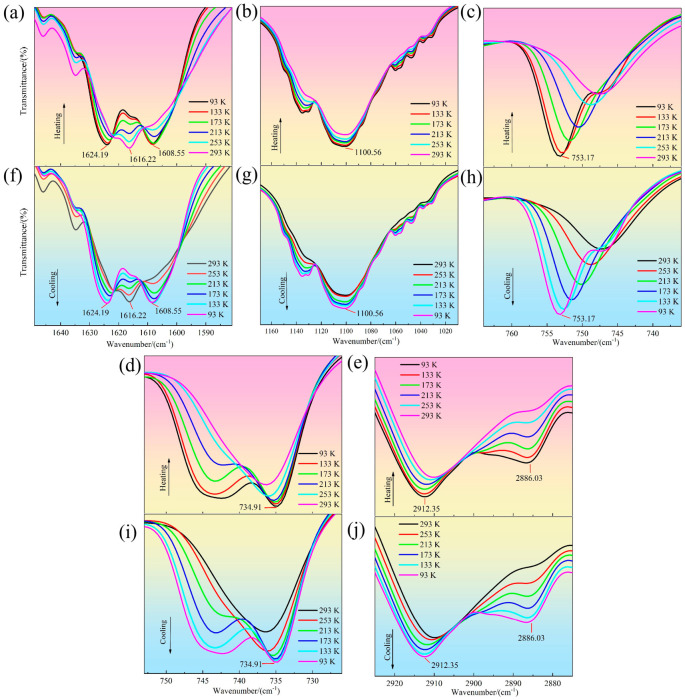
Variabletemperature infrared spectra of compounds **1**–**5**. Cooling (293 → 93 K): (**a**) **1**, (**b**) **2**, (**c**) **3**, (**d**) **4**, and (**e**) **5**; heating (93 → 293 K): (**f**) **1**, (**g**) **2**, (**h**) **3**, (**i**) **4**, and (**j**) **5**.

Compounds **1**–**5** were subjected to variable-temperature infrared measurements at 293, 253, 213, 173, 133, and 93 K. The cooling and heating curves are shown in Figure 4a–j. The spectrum of compound **1** shows bands at 1570–1660 cm^−1^, corresponding to N-H bending vibrations. During heating, the shape of the bands at 1608 and 1624 cm^−1^ gradually transformed from narrow and sharp to broad and blunt. The intensity of the split bands decreased progressively, and the wavenumbers shifted toward higher frequencies. A new peak emerged at 1616 cm^−1^ and gradually gained intensity with increasing temperature. This indicates a change in the C-C-N bond angle, which in turn affects the electron distribution at the N-H bond, leading to a blue-shift in the N-H bending vibration frequency. At low temperatures, the intermolecular interactions in compound **1** form a rigid hydrogen-bond network composed of N-H···O and N-H···N bonds. The two N-H bonds are strongly coupled, resulting in a sharp double peak. During the heating process, lattice expansion increases the interatomic spacing, partially breaking the hydrogen bonds and weakening their strength. The system is gradually transformed into a state where free N–H bonds, weak hydrogen bonds, and strong hydrogen bonds coexist, resulting in a spectroscopic signature characterized by three distinct bands. The spectrum of compound **2** exhibits bands in the 1010–1170 cm^−1^ range, corresponding to the bending-vibration bands of the C–O–C– group in 18-crown-6. During the heating process, the shape of the peak at 1100 cm^−1^ gradually transformed from narrow and sharp to broad and blunt, while the intensity of the split peak decreased progressively and the wavenumber shifted toward higher frequencies. This indicates that the crown ether undergoes significant disorder as the temperature increases. The spectral lines of compound **3** in the wavenumber range of 730–770 cm^−1^ correspond to the out-of-plane rocking vibrations of continuous methylene chains -(CH_2_)_n_- (*n* ≥ 4). During heating, the shape of the peak gradually changed from broad and round to narrow and sharp, accompanied by a blue-shift. The opposite behavior was observed during the cooling process, indicating that the length of the diamine chains in the compound changes with increasing temperature. The spectral lines in the wavenumber range of 730–770 cm^−1^ for compound **4** correspond to the out-of-plane rocking vibrations of continuous methylene chains -(CH_2_)_n_- (*n* ≥ 4). During heating, the shape of the peak gradually changed from broad and rounded to narrow and sharp, accompanied by splitting to form a new peak, as well as a blue-shift. The reverse behavior was observed during the cooling process, indicating that the lengths of the diamine chains in the compound change significantly with increasing temperature. The spectral lines at 2870–2930 cm^−1^ for compound **5** correspond to the stretching vibrations of the -CH_2_- group. During the heating process, the shape of the peak gradually changed from narrow and sharp to broad and blunt; the wavenumbers shifted toward higher frequencies. The bands at 2886 and 2912 cm^−1^ coalesced into a single band. The reverse behavior was observed during the cooling process, indicating that the diamine chain in the compound transitions from an ordered to a disordered state with increasing temperature. Zhang et al. [39] investigated the self-assembly behavior of a DNA-mimetic amphiphilic copolymer, PDCAI, and its molecular-recognition interaction with thymine via variable-temperature infrared spectroscopy (hydrogen bonding). During variable-temperature scanning, the C=O stretching vibration peak was red-shifted, and the N-H stretching vibration peak was blue-shifted, confirming the presence of hydrogen bonds. By monitoring the gradual disappearance or reemergence of the characteristic bands of hydrogen bonding as the temperature increased, the stability of the hydrogen bonds was qualitatively evaluated. For compound **3**, which undergoes a structural phase transition, the methylene out-of-plane wagging vibration peak at 730–770 cm^−1^ gradually becomes sharper and shifts continuously toward higher wavenumbers as the temperature increases within the phase transition range of 250–280 K. This change directly corresponds to the increase in unit cell symmetry, the change in the direction of one-dimensional chain extension, and the continuous adjustment of the crown ether dihedral angle during the phase transition. For compound **5**, which also undergoes a structural phase transition, within the phase transition range of 270–300 K, the methylene stretching vibration peak at 2870–2930 cm^−1^ gradually merges and shifts continuously toward lower wavenumbers. This phenomenon is closely related to the order–disorder transition of the hexamethylenediamine cation and the change in the propagation direction of the one-dimensional chain. In contrast, compounds **1**, **2**, and **4** did not exhibit characteristic spectral discontinuities or rearrangements associated with crystalline phase transitions; their infrared peaks varied with temperature due to thermally induced molecular dynamic relaxation, without accompanying crystalline structural phase transitions, and remained structurally stable within the tested temperature range.

### 3.2. Variable-Temperature XRD Analyses of Compounds ***1***–***5***

#### Variable-Temperature XRD Patterns of Compounds **1**–**5** (a), (b), (c), (d), and (e)

**Figure 5:**
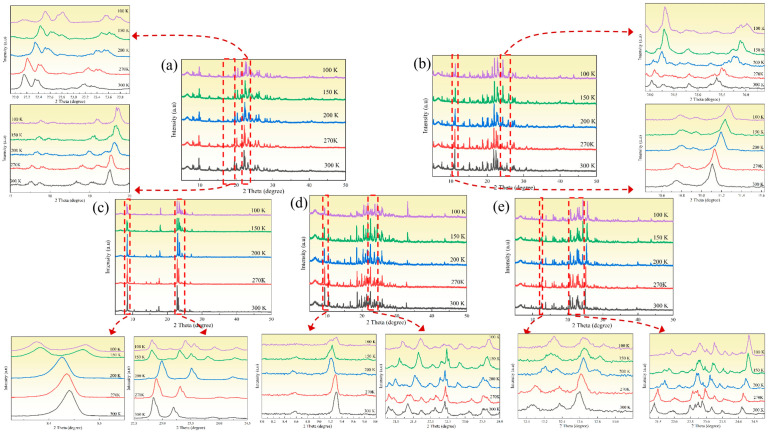
Variable-temperature XRD patterns of (**a**) compound **1**, (**b**) **2**, (**c**) **3**, (**d**) **4**, and (**e**) **5**. Enlarged views of the peak-changing regions are shown as insets.

Variable-temperature XRD analyses of compounds **1**–**5** were performed to further verify the occurrence of structural phase transitions. The XRD peaks of compounds **1**, **2**, and **4** were similar within the temperature range of 100–300 K (Figure 5). However, at different temperatures, the intensity of the diffraction peaks varied slightly, and their positions shifted, indicating continuous thermal expansion of the material. Furthermore, the crystal structure remained stable within the measured temperature range without any structural phase transitions. For compound **3**, the two smaller diffraction peaks in the range of 8.25–8.7° abruptly coalesced into a larger peak when the temperature was increased to 200–300 K. The peak at 22.5–24.5° was shifted, with a certain degree of splitting. These results indicate that the structure undergoes a transition from low symmetry to high symmetry with increasing temperature, suggesting that compound **3** undergoes a structural phase transition. The diffraction peaks of compound **5** at 11.9–13.2° split when the temperature was raised to 200–300 K, and the intensity of the peak also changed. The diffraction peaks at 21–25° were displaced, with a certain degree of splitting, and the intensity changed. These results indicate that compound **5** undergoes a structural phase transition. Therefore, the changes in the diffraction peaks indicate structural alterations in compounds **3** and **5**, consistent with the results from the DSC analysis, thereby further confirming the structural phase transition in compounds **3** and **5**.

### 3.3. Analysis of Intermolecular Interactions of Compounds ***1***–***5***

**Figure 6:**
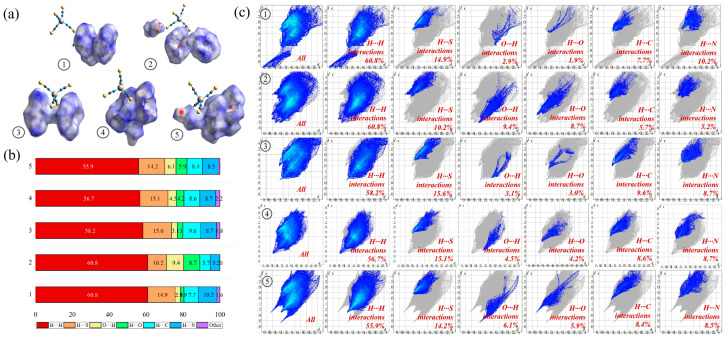
Hirshfeld surfaces (**a**), fingerprint plots with labeled interaction percentages (**c**), and a bar chart of percentage contributions (**b**) for compounds **1**–**5**.

Because of the unique nature of diamine organic molecules, hydrogen bonding is the primary intermolecular force within these compounds. To investigate the influence of hydrogen bonding interactions on the structure and properties of compounds **1**–**5**, the weak interactions in these five compounds were evaluated in detail using the CrystalExplorer 17 software by employing the CIF files [40]. The Hirshfeld surfaces were generated for the sandwich-type crown ether–diammonium supramolecular dication structure unit (i.e., the (C_2_H_2n+6_N_2_)^2+^ cation with two 18-crown-6 molecules attached via N–H···O hydrogen bonds), as this is the structural moiety responsible for supramolecular recognition and assembly. The inorganic [Mn(NCS)_4_]^2−^ anions were treated as surrounding species that interact with the organic cation primarily through C–H···S and N–H···S contacts. As shown in Figure 6, different regions on the Hirshfeld surface represent distinct weak interactions: the red, blue, and white regions indicate hydrogen bonds, van der Waals forces, and extremely weak intermolecular interactions that can be considered negligible, respectively. The red regions are primarily concentrated near the nitrogen atoms of the diamines, which, however, are not directly exposed to the environment because the diamine N–H groups are engaged in hydrogen bonding with the oxygen atoms of the crown ethers. Therefore, the N–H···O interactions are largely intramolecular within the organic cation unit. The intermolecular interactions between the organic cation and the surrounding [Mn(NCS)_4_]^2−^ anions mainly occur through C–H···S hydrogen bonds (from the crown ether methylene groups to the thiocyanate sulfur atoms) and, to a lesser extent, N–H···S contacts where the diamine N–H bonds point toward the anions. The quantitative contributions of various intermolecular contacts to the Hirshfeld surface are summarized in Figure 6b. For compounds **1**–**5**, the dominant interaction is always H···H contacts, which gradually decreased from 60.8% in compound **1** to 60.8%, 58.2%, 56.7%, and 55.9% in compounds **2**, **3**, **4**, and **5**, respectively. The N···H/H···N contacts (which include both N–H···O and N–H···S hydrogen bonds) are strongest in compound **1** (ca. 10.2%) due to the additional acetonitrile molecule, while in compounds **2**–**5** they range from 5.2% to 8.7%. The C···H/H···C contacts (mainly from crown ether methylene groups to thiocyanate sulfur atoms via C–H···S) are more pronounced in compounds with longer diamine chains, consistent with the increased flexibility and more extensive C–H···S networks (Tables S8–S12). Similarly, Balakrishnan et al. [41] used the CrystalExplorer program to perform a quantitative analysis of the hydrogen-bond network in the supramolecular eutectic of 18-crown-6, confirming the effectiveness of this method in elucidating intermolecular interactions and the relationship between structure and properties.

Hirshfeld surface analysis indicates H···H van der Waals contacts dominate crystal packing, while N–H···O intramolecular hydrogen bonds and C–H···S cation–anion hydrogen bonds dominate supramolecular orientation and phase transition. With the increase in alkyl chain length, the proportion of H···H interactions gradually decreases, while C–H···S contributions slightly rise, reflecting enhanced conformational flexibility and more extensive weak hydrogen-bond networks. This trend is well correlated with thermal and dielectric behaviors.

### 3.4. Thermal Analysis of Compounds ***1***–***5***

**Figure 7:**
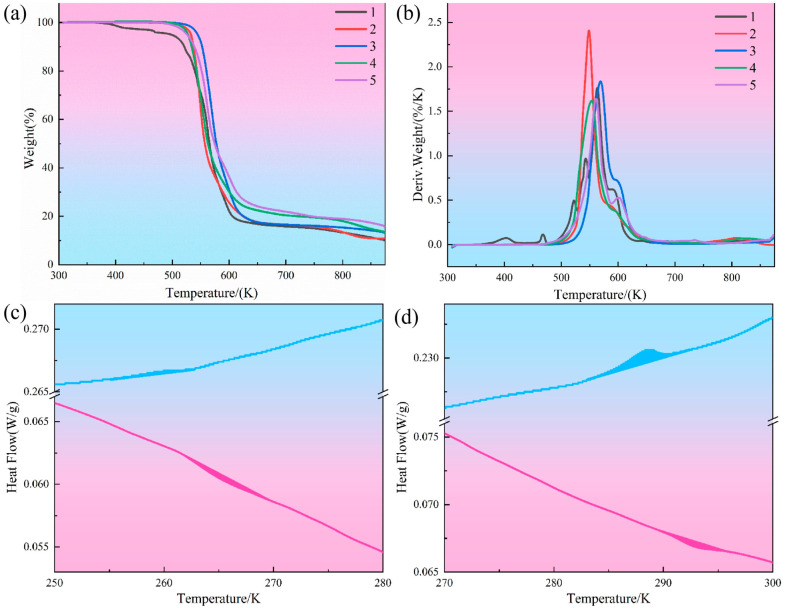
(**a**) TG diagram of compounds **1**–**5,** (**b**) DTA diagram of compounds **1**–**5,** (**c**) DSC diagram of compound **3,** (**d**) DSC diagram of compound **5**.

Thermogravimetric analysis (TGA) within the temperature range of 300–850 K was performed at a heating rate of 10 K/min under nitrogen as an inert gas using approximately 8 mg of sample. As shown in Figure 7a,b, compound **1** undergoes mass loss in five distinct stages owing to the presence of one molecule of acetonitrile within its crystal structure. Compound **1** first loses one molecule of acetonitrile. Subsequent mass loss proceeds in a manner similar to that in the other compounds: firstly, the crown ether molecules in the crystal structure are lost, followed by the organic diamine, and finally, the inorganic components undergo decomposition. Therefore, compound **1** is the least thermally stable, where decomposition begins at 381 K. Decomposition of compounds **2**, **3**, **4**, and **5** is initiated at 508, 511, 498, and 491 K, respectively. The analysis indicates that compound **3** exhibits the highest thermal stability. This is likely attributed to the stronger hydrogen bonding interactions in compound **3**. To substantiate the attribution of this higher thermal stability to stronger hydrogen bonding interactions, we directly compared the key hydrogen bond parameters of compound **3** with those of compounds **1**, **2**, **4**, and **5** (Tables S8–S12). The average N–H···O hydrogen bond length in compound **3** is 2.88 Å (ranging from 2.85 to 2.98 Å), which is shorter than those in compound **2** (2.91 Å), compound **4** (2.92 Å), and compound **5** (2.91 Å). Compound **1** exhibits a comparably short average N–H···O bond length (2.86 Å), but its overall thermal stability is compromised by the early loss of the acetonitrile solvent molecule. Furthermore, the average C–H···S hydrogen bond length in compound **3** (3.55 Å) is shorter than those in compound **2** (3.64 Å), compound **4** (3.73 Å), and compound **5** (3.76 Å). These quantitative comparisons indicate that compound **3** possesses a stronger and more extensive hydrogen-bonding network, which accounts for its superior thermal stability. A detailed analysis of the thermal stability of compounds **1**–**5** is presented in Figure S9.

Figure 7c shows the DSC curve of compound **3** within the temperature range of 250–280 K, characterized by an exothermic peak at 260 K during cooling and a distinct endothermic peak at 265 K during heating. Compound **3** exhibits a phase transition near 260 K. The DSC curve of compound **5** (Figure 7d) within the temperature range of 270–300 K exhibits an exothermic peak at 288 K during the cooling process and a distinct endothermic peak at 293 K during the heating process. The data indicate that compound **5** undergoes a phase transition near 290 K. Such structural phase transitions driven by order–disorder changes in crown ethers and organic cations have been widely reported in crown-ether-based supramolecular clathrates [42]. The test results are consistent with the variable-temperature XRD and infrared spectroscopy data, as well as the potential energy for molecular rotation.

### 3.5. Analysis of Dielectric Properties of Compounds ***1***–***5***

**Figure 8:**
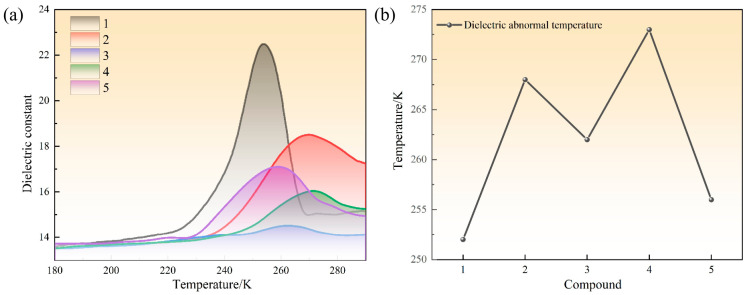
(**a**) Dielectric anomaly curves of compounds **1**–**5** in the a-axis direction at a frequency of 1 kHz; (**b**) dielectric anomaly temperature of compounds **1**–**5**.

The dielectric properties are influenced by factors such as the frequency, temperature, and surface roughness, which are vital in analyzing the material. Compounds **1**–**5** with superior crystal structures were selected for fabricating capacitors using conductive silver paste and copper wires. Dielectric tests were conducted within the frequency range from 500 Hz to 10 kHz (as shown in Figures S12–S16). Figure 8a shows the dielectric anomaly curves of compounds **1**–**5** at 1 kHz. Compounds **1**, **2**, **3**, **4**, and **5** exhibit dielectric anomaly peaks at 252, 268, 262, 273, and 255 K, respectively. Overall, the dielectric constants of compounds **1**–**5** increased gradually with increasing temperature, decreased rapidly after reaching their maximum values, and finally stabilized. It should be noted that only compounds **3** and **5** undergo structural phase transitions accompanied by changes in crystal system and space group, as confirmed by variable-temperature single-crystal X-ray diffraction. For compounds **1**, **2**, and **4**, no such symmetry breaking is observed. Instead, their dielectric anomalies arise from different local mechanisms: Compound **1** contains one acetonitrile molecule; as a polar small molecule, its orientational changes with temperature may induce a dielectric relaxation response. Compound **2** shows partial disorder of crown ethers at low temperature and complete disorder at room temperature, as revealed by crystal structure analysis; this local dynamic disorder is sufficient to produce a dielectric anomaly. Compound **4** exhibits temperature-dependent conformational changes in the diamine chain, as evidenced by variable-temperature infrared spectroscopy, which may also contribute to the dielectric response.

As shown in Figure 8b, the temperature at which the dielectric anomaly peaks occur exhibits a systematic dependence on the diamine chain length. For compounds with even-numbered carbon chains (*n* = 2, 4, 6), the anomaly temperatures are 252 K (compound **1**), 262 K (compound **3**), and 255 K (compound **5**), whereas for odd-numbered chains (*n* = 3, 5), they are 268 K (compound **2**) and 273 K (compound **4**). This odd–even alternation cannot be explained solely by chain-length-dependent steric hindrance or intermolecular interaction strength. Instead, it correlates well with the dihedral angle between the two crown ether planes (Figure 1): when the crown ethers adopt a nearly parallel arrangement (even n, compounds **1**, **3**, **5**), the dielectric anomaly occurs at relatively lower temperatures; when they form a pronounced angle (odd n, compounds **2**, **4**), the anomaly shifts to higher temperatures. We propose that the parallel orientation facilitates cooperative molecular polarization and dipole reorientation under an alternating electric field, requiring less thermal energy to trigger the dielectric response. In contrast, the angled arrangement imposes geometrical constraints that hinder polarization, necessitating higher temperatures to activate molecular motion. This interpretation is consistent with the structural data (Tables S8–S12) showing that hydrogen-bonding networks and packing modes differ significantly between even- and odd-chain compounds. Thus, the dielectric behavior is governed not simply by chain length but by the crown ether dihedral angle dictated by the parity of the diamine chain, which modulates the ease of molecular polarization and reorientation.

### 3.6. UV–Vis Absorption Spectroscopy and Density of States Analysis

**Figure 9:**
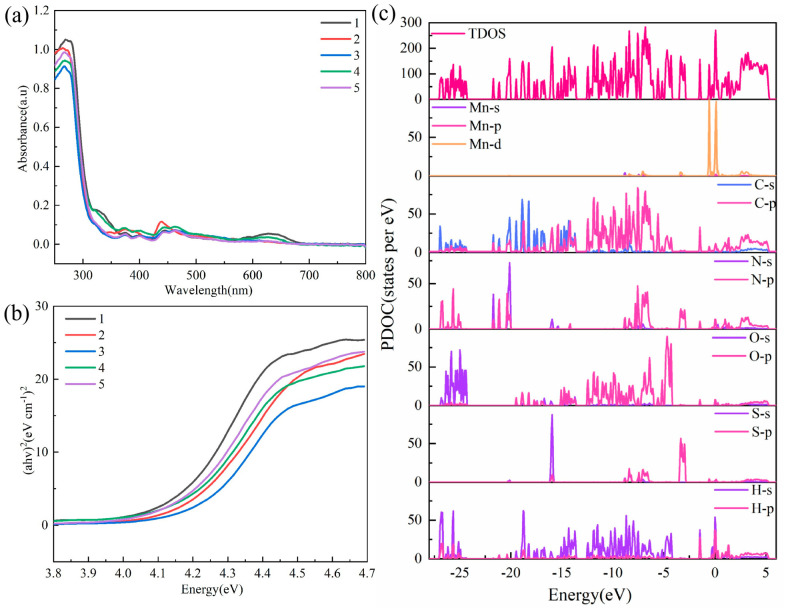
(**a**) UV–vis absorption spectra of compounds **1**–**5**; (**b**) Tauc plots for compounds **1**–**5**; (**c**) density of states diagram of compound **1**.

Compounds **1**–**5** exhibited wide-bandgap insulator behavior. The bandgaps were calculated from the solid-state ultraviolet-visible (UV–vis) absorption spectra. Solid-state UV diffuse reflectance spectra of compounds **1**–**5** were acquired at room temperature (Figure 9), demonstrating maximum absorptions at approximately 268, 263, 267, 266, and 265 nm, respectively (Figure S17). From the Tauc curves, the optical bandgaps of compounds **1**–**5** were determined as 4.09, 4.11, 4.14, 4.09, and 4.11 eV, respectively. The bandgaps of BaTiO_3_, Gr_2_O_3_**,** and MnO (greater than 3.2 eV) are also shown. The bandgaps of the supramolecular host–guest complex are primarily determined by the anionic metal complex [Mn(NCS)_4_]^2−^ and thus remain similar. Minor differences arise from the organic cations. For comparison, Yuwei et al. [43] reported bandgaps of 2.75 eV and 2.88 eV for two isostructural materials containing different organic cations. No further mechanistic interpretation is attempted here, as the present compounds are not hybrid perovskites and do not possess an extended inorganic framework.

To further analyze the wide-bandgap insulator properties of compounds **1**–**5**, the energy gap distribution was investigated by comparing the total density of states (DOS) with the partial density of states (PDOS) (as shown in Figure S18). Taking compound **1** as an example (Figure 9c), the 2p orbitals of the N atoms predominantly occupy the energy band at the top of the valence band. The energy band at the bottom of the conduction band primarily originates from the 4d orbitals of the Mn atoms. The minimum point of the conduction band and the maximum point of the valence band primarily originate from the electronic states of the Mn and N atoms. Similarly, those of compounds **2**–**5** are mainly derived from the electronic states of the Mn and N atoms, further indicating that the optical bandgap of the material depends on the inorganic framework.

### 3.7. Magnetic Analysis of Compounds ***1***–***5***

**Figure 10:**
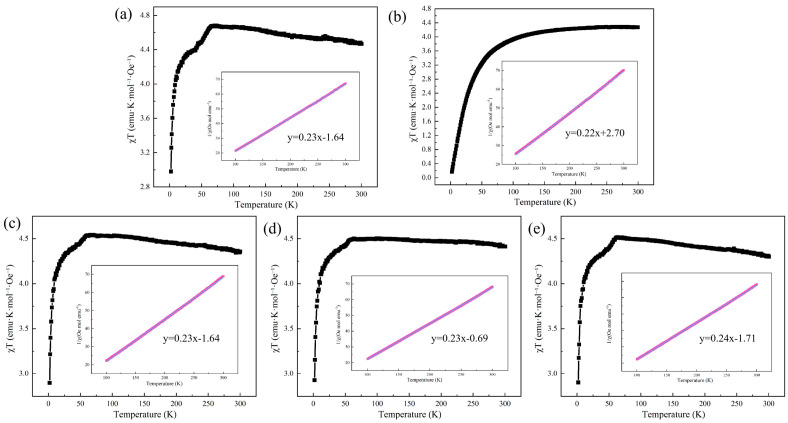
Temperature-dependent magnetic susceptibility plots for compounds **1**–**5** (**a**–**e**). Main plot: χT versus T; Inset: 1/χ versus T with Curie–Weiss fitting in the high-temperature region.

Under an applied magnetic field of 1000 Oe, the temperature-dependent magnetic susceptibility of compounds **1**–**5** was measured over the range of 2–300 K. The results are presented as χT–T and 1/χ–T curves (Figure 10). The χT–T curves provide a visual indication of the type of magnetic interaction, while the 1/χ–T curves are used for Curie–Weiss fitting, a standard method for characterizing magnetic behavior [44,45,46].

At 300 K, the χT values for compounds **1**–**5** were 4.47, 4.27, 4.36, 4.41, and 4.30 emu·K·mol^−1^·Oe^−1^, respectively. All of these values are in excellent agreement with the theoretical value of 4.375 emu·K·mol^−1^·Oe^−1^ for isolated high-spin Mn^2+^ (S = 5/2, g = 2.0), demonstrating that the system’s magnetic properties primarily originate from the high-spin Mn^2+^ centers in the inorganic framework.

During the cooling process from 300 K to 50 K, the χT values of compounds **1**, **3**, **4**, and **5** remained essentially constant; when the temperature fell below 50 K, χT exhibited a gradual decrease with decreasing temperature. This behavior is a typical feature of weak ferromagnetic interactions superimposed on the zero-field splitting effect at low temperatures. In sharp contrast, compound **2** exhibits a sharp decrease in χT below 50 K, which is typical of antiferromagnetic coupling behavior.

The magnetic susceptibility data in the high-temperature range (100–300 K) follow the Curie–Weiss law: *χ* = *C*/(*T* − *θ*). Here, *C* is the Curie constant and θ is the Tussauds temperature. The key magnetic parameters obtained by linear fitting the 1/χ–T curve are listed in Table S13. The fitting results indicate that: The Weyss temperatures θ of compounds **1**, **3**, **4**, and **5** are all positive (+7.1862, +7.0116, +3.0217, and +7.2197 K), clearly confirming the presence of weak ferromagnetic interactions in the system; the Weiss temperature θ of compound **2** is negative (−12.0849), which is a direct indication of antiferromagnetic interactions. The effective magnetic moments μ_eff_ calculated at room temperature were 5.9237, 5.9839, 5.8514, 5.9297, and 5.8176 μ_B_, respectively, all of which are consistent with the theoretical magnetic moment of high-spin Mn^2+^ (≈5.92 μ_B_).

**Figure 11:**
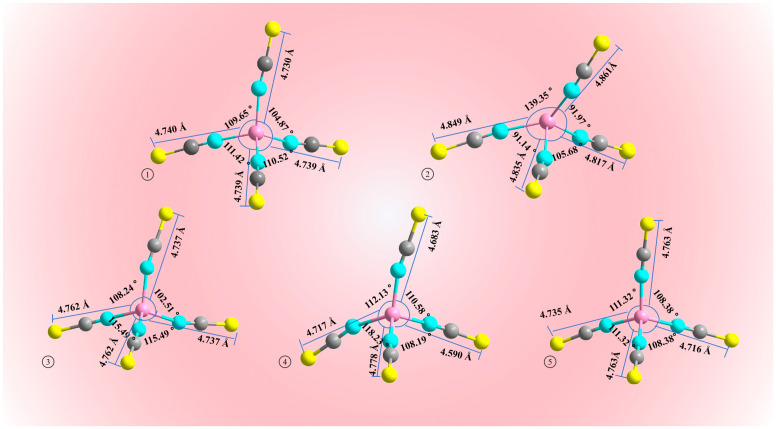
Diagrams of inorganic anions in compounds **1**–**5**.

The significant magnetic differences observed among compounds **1**–**5** stem fundamentally from the differences in the local coordination environment and geometric configuration of the inorganic anion unit [Mn(NCS)_4_]^2−^. In compounds **1**, **3**, **4**, and **5**, the [Mn(NCS)_4_]^2−^ coordination unit maintains a relatively regular tetrahedral configuration (Figure 11), with relatively uniform distributions of Mn–N bond lengths and N–Mn–N bond angles; the overall bond lengths are moderate and show no significant distortion. This highly symmetric, low-distortion coordination environment weakens the antiferromagnetic superexchange channel and is more conducive to the transfer of weak ferromagnetic coupling between molecules; therefore, the system exhibits weak ferromagnetic behavior.

In stark contrast, the [Mn(NCS)_4_]^2−^ unit in Compound **2** exhibits significant tetrahedral distortion: on the one hand, the Mn–NCS bond lengths are markedly elongated; on the other hand, the coordination angles show extreme deviations, forming two vastly different angles of 139.35° and 91.97°, which severely disrupt the ideal tetrahedral structure. This severe tetrahedral distortion of [Mn(NCS)_4_]^2−^, together with crown ether dynamic disorder, enhances antiferromagnetic superexchange pathways mediated by SCN^−^ ligands. Consequently, Compound **2** exhibits antiferromagnetic properties that are entirely distinct from those of the other four compounds. Cs_3_Mn_2_O_4_, synthesized by Nuss et al. [47], exhibits similar antiferromagnetic properties, arising primarily from strong antiferromagnetic coupling between the Mn^2+^ and Mn^3+^ ions.

### 3.8. Analysis of Electrochemical Properties of Compounds ***1***–***5***

Cyclic voltammetry (CV) is an important electrochemical research method. By analyzing the CV curve, important parameters for studying electrode processes, reaction mechanisms, and the kinetics of electrode reactions can be obtained, such as the peak potentials of the anode and cathode (*E*_pc_ and *E*_pa_, respectively), their difference (Δ*E*_p_), and the ratio of the peak currents (*i*_pc_/*i*_pa_). For reversible Nernstian electrode reaction systems, *i*_pc_ and *i*_pa_ are essentially equal, i.e., *i*_pc_/*i*_pa_ ≈ 1, and *E*_pc_ and *E*_pa_, as well as their difference Δ*E*_p_, are independent of the scanning rate *υ*.

$$\Delta E_{p}=E_{\mathrm{pa}}-E_{\mathrm{pc}}=2.3\frac{RT}{nF}\approx \frac{59}{n}\left(\mathrm{mV}\right)$$

$$E_{1/2}=\frac{E_{\mathrm{pa}}+E_{\mathrm{pc}}}{2}$$

However, because most electrode reactions are irreversible or involve irreversible steps, the shape of the corresponding CV curves typically deviates from the “duckbill” profile. For quasi-reversible systems, |*i*_pc_| ≠ |*i*_pa_|, and |Δ*E*_p_| is not only larger than that of reversible systems but also increases with increasing *υ*. The magnitude of Δ*E*_p_ and its variation with temperature are commonly used as important criteria for determining whether an electrode reaction is reversible, as well as the degree of reversibility. If |Δ*E*_p_| ≈ 2.3 *RT*/n*F* and does not vary with *υ*, the reaction is reversible; if |Δ*E*_p_| > 2.3 *RT*/n*F* and increases with increasing *υ*, the reaction is irreversible [48].

**Figure 12:**
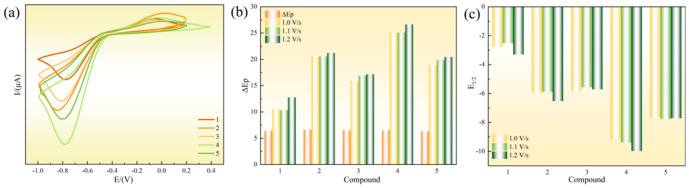
(**a**) Cyclic voltammograms of compounds **1**–**5** at 1.0 V/s; (**b**) Δ*E*_p_ diagrams of compounds **1**–**5** at different scan rates; (**c**) *E*_1_/_2_ diagrams of compounds **1**–**5** at different scan rates.

Table S14 shows that the |Δ*E*_p_| values for compounds **1**–**5** all exceed 2.3 $\frac{RT}{nF}$ and increase with *v*. Because |*i*_pa_| ≠ |*i*_pc_|, compounds **1**–**5** are all quasi-reversible systems. Based on the data from Table S14, Δ*E*_p_ (Figure 12b) and *E*_1/2_ (Figure 12c) were plotted for compounds **1**–**5**. As shown in the figures, compounds **1**–**5** all exhibit excellent cycling stability and electrochemical properties. The |Δ*E*_p_| values demonstrate regular fluctuations that perfectly correlate with the changes in the dihedral angle of the crown ether. Both patterns exhibit an alternating trend of “increase → decrease → increase again → decrease again”. Note that the dihedral angle between the planes of the two crown ether molecules (as defined in Figure 1) was largest for compounds **2** and **4**, and the corresponding |Δ*E*_p_| values were also the largest. *E*_1/2_ decreased with increasing length of the diamine chain, indicating that the reduction reaction occurs at a more negative potential and the reduced state exhibits enhanced stability. However, the periodicity of the compounds remained regulated by changes in the dihedral angle of the crown ether. The data reveal that Alkyl chain length overall regulates electrochemical response, while crown ether interplanar dihedral angle acts as the key structural factor controlling electron transfer kinetics. The synergistic interaction between these two elements governs the periodic variation in the electrochemical behavior.

## 4. Conclusions

Five novel aliphatic diamine manganese thiocyanate coordination compounds bearing alkyl chains (**1**–**5**) were systematically synthesized, and their crystal structures, thermoresponsive behavior, and physicochemical properties were characterized, revealing core structure–property relationships. Upon lengthening the carbon chain of the diamine from ethylenediamine to hexamethylenediamine, the diamine chain twists and folds between the two crown ether molecules. This causes the dihedral angle between the crown ether planes to exhibit a distinct pattern of even and odd variations. Chains with an even number of carbon atoms tend to arrange themselves in parallel, while chains with an odd number of carbon atoms form a distinct angle. This structural pattern directly governs the macroscopic phase transition in the crystal, further inducing an even–odd periodicity in the dielectric and electrochemical responses of the material. When the number of carbon atoms in the diamine is even, the dielectric anomaly temperature is lower and the |Δ*E*_p_| value is smaller; when odd, the dielectric anomaly temperature is higher and the |Δ*E*_p_| value increases significantly. The wide-bandgap insulator properties and magnetic behavior are primarily influenced by the inorganic coordination units within the system, where compound **2** exhibits distinct antiferromagnetic characteristics. This study highlights the significance of structural regulation in designing multifunctional material properties, providing a clear molecular engineering approach for developing structurally tunable organic–inorganic hybrid materials with predictable performance.

## Data Availability

The original contributions presented in this study are included in the article/Supplementary Material. Further inquiries can be directed to the corresponding authors.

## Supplementary Materials

The following supporting information can be downloaded at: https://www.mdpi.com/article/10.3390/molecules31122012/s1, Figure S1: Synthesis of compounds **1**–**5**; Figure S2: Minimal components of compounds **1**–**5** at LT (a) and RT (b); Figure S3: Diamine diagrams of compounds **1**–**5** at LT and RT; Figure S4: One-dimensional chain diagrams of compounds **1**–**5** at LT (a–e); Figure S5: One-dimensional chain diagrams of compounds **1**–**5** at RT (a–e); Figure S6: 2D hydrogen-bonding networks of compounds **1**–**5** at LT (a) and RT (c); side lengths of quadrilaterals constructed with manganese atoms as vertices. The longer sides are designated as “a” and the shorter sides as “b”. The plot presents the measured a and b values for compound **1**–**5** at LT (b) and RT (d); Figure S7: Stacking diagrams of compounds **1**–**5** at LT (a–e), and at RT (f–j). The blue polyhedron represents the inorganic anion [Mn(NCS)_4_]^2−^ unit, while the organic diamine–crown ether cationic framework is depicted as a wireframe model, clearly illustrating the changes in supramolecular assembly and hydrogen-bond networks at different temperatures; Figure S8: IR spectra of compounds **1**–**5** (a–e); Figure S9: DSC diagram of compounds **1**, **2**, **4** (a–c); Figure S10: TG of compounds **1**–**5** (a–e); Figure S11: XRD patterns of compounds **1**–**5** (a–e); Figure S12: The dielectric constant curves of compound **1** in the a, b and c axis directions respectively (a–c); Figure S13: The dielectric constant curves of compound **2** in the a, b and c axis directions respectively (a–c); Figure S14: The dielectric constant curves of compound **3** in the a, b and c axis directions respectively (a–c); Figure S15: The dielectric constant curves of compound **4** in the a, b and c axis directions respectively (a–c); Figure S16: The dielectric constant curves of compound **5** in the a, b and c axis directions respectively (a–c); Figure S17: The solid UV spectra of compounds **1**–**5** (a–e); Figure S18: The density of states of compounds **1**–**5** (a–e); Figure S19: Cyclic voltammograms of compounds **1**–**5** (a–e); Table S1: Crystallographic data of compound **1**–**5**; Table S2: The partial bond length (Å) and bond angle (°) of compound **1** at 100 K and 293 K were obtained; Table S3: The partial bond length (Å) and bond angle (°) of compound **2** at 100 K and 293 K were obtained; Table S4: The partial bond length (Å) and bond angle (°) of compound **3** at 100 K and 293 K were obtained; Table S5: The partial bond length (Å) and bond angle (°) of compound **4** at 100 K and 293 K were obtained; Table S6: The partial bond length (Å) and bond angle (°) of compound **5** at 100 K and 293 K were obtained; Table S7: The distance between two N atoms in diamine ions in compounds **1**–**5**; Table S8: Some hydrogen bond parameters of compound **1** at 100 K and 293 K; Table S9: Some hydrogen bond parameters of compound **2** at 100 K and 293 K; Table S10: Some hydrogen bond parameters of compound **3** at 100 K and 293 K; Table S11: Some hydrogen bond parameters of compound **4** at 100 K and 293 K; Table S12: Some hydrogen bond parameters of compound **5** at 100 K and 293 K; Table S13: Compounds **1**–**5** Magnetic data; Table S14: Electrochemical data of compounds **1**–**5**.
