# Supplementary material for: Design and Control of Supramolecular Structure in Crown Ether–Manganese Thiocyanate Complexes Tuned by Aliphatic Diamine Alkyl Chains: Parity-Dependent Modulation of Dielectric and Electrochemical Properties
Source: Molecules. 2026 Jun 9;31(12):2012. doi: 10.3390/molecules31122012 (PMC13306040; doi:10.3390/molecules31122012)
Supplement: Supplementary file 1 [file molecules-31-02012-s001.zip › molecules-4240327-supplementary.pdf]

Supporting Information

Design and Control of Supramolecular Structure in Crown Ether–Manganese Thiocyanate Complexes Tuned by Aliphatic Diamine Alkyl Chains: Parity-Dependent Modulation of Dielectric and Electrochemical Properties

Tong Zhang [1,2], Hongzhi Hu [1,2,*], Adila Abuduheni [1,2], Yang Liu [1,2,3,*] and Zunqi Liu [1,2,3,*]

1 Chemistry and Chemical Engineering College, Xinjiang Agricultural University, Urumqi 830052, China; tongzhang070300@163.com (T.Z.); 17799751675@163.com (A.A.)

2 Xinjiang Key Laboratory for High Value Utilization of Agricultural and Livestock By-Products, Urumqi 830052, China

3 Xinjiang Sub-Center National Engineering Research Center of Novel Equipment for Polymer Processing, Urumqi 830052, China

* Correspondence: huhongzhi305@163.com (H.H.); ly2021@xjau.edu.cn (Y.L.); lzq@xjau.edu.cn (Z.L.)

Sample synthesized

In this study, manganese chloride tetrahydrate, ethylenediamine hydrochloride, 1,3-propanediamine, 1,4-butanediamine dihydrochloride, 1,5-pentanediamine hydrochloride, 1,6-hexanediamine hydrochloride, potassium thiocyanate, 18-crown-6 were all purchased from Shanghai Jinjinle Co., Ltd., which were commercially available analytically pure products. Hydrochloric acid and acetonitrile were purchased from Tianjin Yongsheng Fine Chemical Co., Ltd., which were commercially available analytically pure products without further purification before use.

1. Synthesis of $(C_2H_{10}N_2)^{2+}$(18-crown-6)$_2$$[Mn(NCS)_4]^{2-}$$(C_2H_3N)$ (**1**)

0.200 g (1 mmol) $MnCl_2{\cdot}4H_2O$, 0.39 g (4 mmol) KSCN, 0.13 g (1 mmol) ethylenediamine hydrochloride and 0.53 g (1 mmol) 18-crown-6 were dissolved in 20 mL water and acetonitrile mixed solution, respectively. 0.5 mL hydrochloric acid was dropped into the mixed solution, and the mixed solution was stirred continuously during the dropping process. The mixed solution was placed for 10 min, and placed in a cool place, waiting for the solution to evaporate naturally, and light yellow crystals were grown about one week.

2. Synthesis of $(C_3H_{12}N_2)^{2+}$(18-crown-6)$_2$[Mn(NCS)$_4$]$^{2-}$ (**2**)

The synthesis process is the same as that of compound 1, only ethylenediamine hydrochloride was replaced by 1,3-propanediamine (0.07 g, 1 mmol). Light yellow crystal was obtained.

3. Synthesis of $(C_4H_{14}N_2)^{2+}$(18-crown-6)$_2$[Mn(NCS)$_4$]$^{2-}$ (**3**)

The synthesis process is the same as that of compound 1, only ethylenediamine hydrochloride was replaced by 1,4-butanediamine dihydrochloride (0.16 g, 1 mmol). Light yellow crystal was obtained.

4. Synthesis of $(C_5H_{16}N_2)^{2+}$(18-crown-6)$_2$[Mn(NCS)$_4$]$^{2-}$ (**4**)

The synthesis process was the same as that of compound 1, and only ethylenediamine hydrochloride was replaced by 1,5-pentanediamine hydrochloride (0.17g, 1mmol). Light yellow crystal was obtained.

5. Synthesis of $(C_6H_{18}N_2)^{2+}$(18-crown-6)$_2$[Mn(NCS)$_4$]$^{2-}$ (**5**)

The synthesis process is the same as that of compound 1, only ethylenediamine hydrochloride was replaced by 1,6-hexanediamine hydrochloride (0.19 g, 1 mmol). Light yellow crystal was obtained. The synthetic routes of the above five compounds are shown in Figure S1

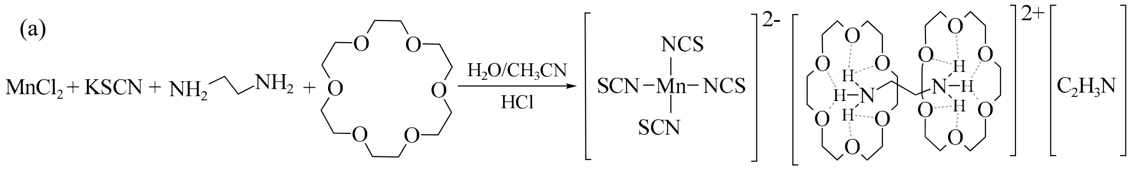

Complex **1**

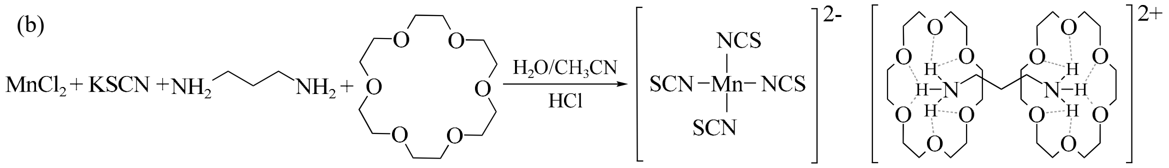

Complex **2**

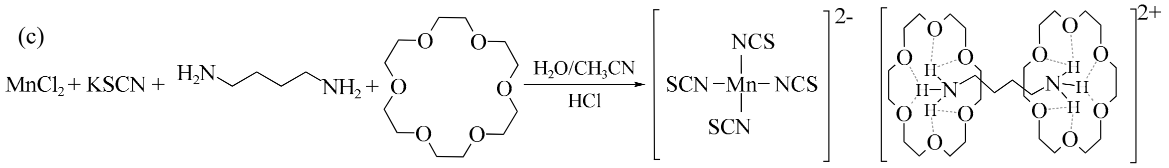

Complex **3**

(d)

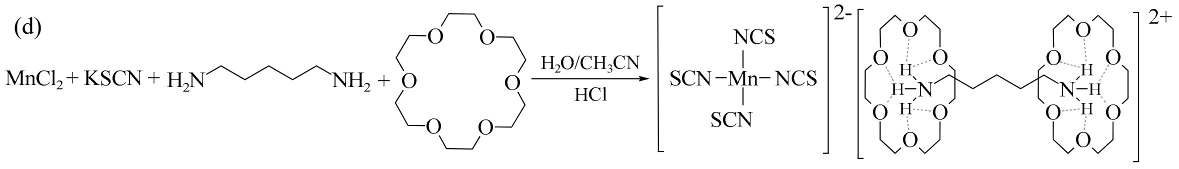

Complex **4**

(e)

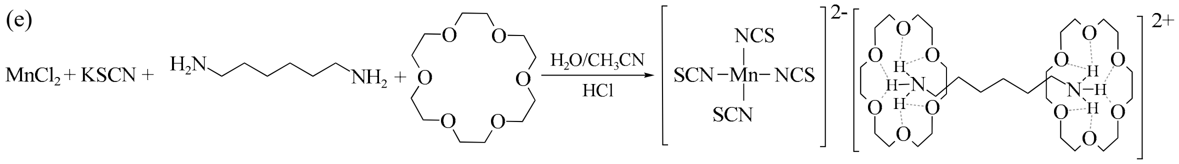

Complex **5**

Figure S1 Synthesis of compounds **1-5**

Experimental equipment and methods

Temperature-dependent infrared spectroscopy (4000-400 cm^{-1}) was determined by FT-IR8700 infrared spectrometer (Shimadzu, Japan), single crystal X-ray diffraction analysis was determined by Bruker Smart Apex II single crystal X-ray diffractometer (Bruker, Germany), and powder X-ray diffraction was determined by powder X-ray diffractometer (Bruker, Germany). Thermogravimetric analysis was performed using the American TAQ50 thermogravimetric analyzer in a nitrogen flow at a heating rate of 10 K / min in the temperature range of 300-850 K. The differential scanning calorimetry (DSC) uses the American TAQ20 instrument to heat and cool the sample under nitrogen protection. The temperature range is 200-300 K, and the heating rate is 10 K / min.The dielectric constant was measured by a TH2828 dielectric constant tester (Changzhou Tonghui Electronics Co., Ltd., China) in the frequency range of 500 Hz to 1 MHz, at a voltage of 1.0 V, and at a heating rate of 2 K / min.UV-visible absorption (200-800 nm) was measured using a Shimadzu UV3600 visible spectrophotometer, and electrochemical tests were performed using a CHI700E electrochemical workstation. The compound sample (70 mg) and carbon black (10 mg) were taken respectively. In the mixed solution of 0.1 mol / L H_2SO_4 and 0.5 mol / L Na_2SO_4, the capacitance voltage characteristic curves (CV curves) at different scanning rates were measured by a three-electrode system, namely glassy carbon electrode, saturated calomel electrode reference electrode and platinum wire electrode.

Determination of crystal structure of compound

Crystals with smooth surface and complete crystal form were selected under the microscope and tested by Bruker Smart Apex II single crystal diffractometer. In the experiments, monochromatic Cu-Kα radiation (λ = 1.54184 Å) was used to scan and collect diffraction data for compound 3 at LT (100 K) and compound 4 at RT (293 K). Mo-Kα radiation (λ = 0.71073 Å) was used to scan and collect diffraction data for the remaining compounds at LT (100 K) and RT (293 K) within a specific angular range. The diffraction points were selected and the structure of the compounds was analyzed by direct method through SHELXTL program. All hydrogen atoms adopt theoretical hydrogen, and the anisotropic temperature factor and the coordinates of non-hydrogen atoms are corrected by the full matrix least squares method. Finally, the crystallographic parameters of compounds **1-5** at 100 K and 293 K were obtained (Table S1).

Table S1 Crystallographic data of compound **1-5**

**Compound**	**1**	**2**	**3**	**4**	**5**
**Chemical formula**	$C_{32}H_{61}MnN_7O_{12}S_4$	$C_{31}H_{60}MnN_6O_{12}S_4$	$C_{32}H_{62}MnN_6O_{12}S_4$	$C_{33}H_{64}MnN_6O_{12}S_4$	$C_{34}H_{66}MnN_6O_{12}S_4$
**Temperature/K**			293		
**Formula weight**	919.05	892.03	906.05	920.08	934.10
**Crystal system**	orthorhombic	triclinic	monoclinic	monoclinic	orthorhombic
**Space group**	*Pnma*	*P-1*	*C2/c*	$P2_1/n$	*Pnma*
a **(Å)**	22.8197(17)	11.7273(7)	21.508(2)	12.8120(12)	24.663(2)
b **(Å)**	14.6192(9)	11.7655(5)	12.2104(12)	18.6685(15)	14.4855(14)
c **(Å)**	13.9377(11)	17.6374(9)	19.609(2)	21.4317(17)	14.0493(13)
α **(°)**	90	108.552(4)	90	90	90
β **(°)**	90	91.421(5)	110.368(12)	105.364(9)	90
γ **(°)**	90	91.703(4)	90	90	90
V **($Å^3$)**	4649.7(6)	2304.6(2)	4827.9(9)	4942.9(8)	5019.2(8)
Z	4	2	4	4	4
**D_{calc} (g·cm^{-1})**	1.313	1.285	1.247	1.236	1.236
***F*(000)**	1948	946	1924	1956	1988
**μ (mm^{-1})**	0.523	0.525	0.502	4.222	0.485
**Measured 2Θ range (°)**	4.038- 49.998	4.174 - 59.148	4.124-49.998	6.38 - 133.19	4.038- 50
**R_{int}**	0.0329	0.0298	0.0418	0.0417	0.0404
**R (I > 2(I)) [a]**	0.0948	0.0652	0.1008	0.1128	0.0762
**wR (all data) [b]**	0.3023	0.1700	0.3466	0.2804	0.2209
**GOF**	1.116	1.022	1.031	1.066	1.099
**CCDC**	2534435	2534437	2537286	2537324	2537335

**Compound**	**1**	**2**	**3**	**4**	**5**
**Chemical formula**	$C_{32}H_{61}MnN_7O_{12}S_4$	$C_{31}H_{60}MnN_6O_{12}S_4$	$C_{64}H_{124}Mn_2N_{12}O_{24}S_8$	$C_{33}H_{62}MnN_6O_{12}S_4$	$C_{34}H_{66}MnN_6O_{12}S_4$
**Temperature/K**			100		
**Formula weight**	919.05	892.03	1812.10	918.06	934.10
**Crystal system**	orthorhombic	triclinic	triclinic	monoclinic	monoclinic
**Space group**	*Pnma*	*P*-1	*P*-1	$P2_1/n$	$P2_1/c$
a **(Å)**	22.8197(17)	11.4707(9)	12.2956(13)	13.0612(8)	14.3767(17)
b **(Å)**	14.6192(9)	11.6138(7)	19.4154(12)	18.4269(8)	13.6354(9)
c **(Å)**	13.9377(11)	17.5560(14)	21.0682(12)	20.2547(11)	24.7007(18)
α **(°)**	90	93.124(6)	100.516(5)	90	90
β **(°)**	90	107.604(7)	91.465(6)	105.641(6)	92.218(9)
γ **(°)**	90	92.318(6)	107.697(7)	90	90
V **($Å^3$)**	4649.7(6)	2222.0(3)	4693.3(7)	4694.3(5)	4838.5(7)
Z	4	2	2	4	4
**D_{calc} (g·cm^{-1})**	1.313	1.333	1.282	1.299	1.282
***F*(000)**	1948	946	1924	1948	1988
**μ (mm^{-1})**	0.523	0.545	4.439	0.518	0.503
**Measured 2Θ range (°)**	4.038-49.998	4.138 - 49.996	4.282- 133.2	4.01 - 49.998	4.118 - 50.084
**R_{int}**	0.0515	0.0293	0.1162	0.0610	0.0443
**R (I > 2(I)) [a]**	0.0537	0.0473	0.1386	0.0594	0.1023
**wR (all data) [b]**	0.1290	0.1169	0.4128	0.1610	0.2985
**GOF**	1.048	1.035	1.041	1.037	1.059
**CCDC**	2534434	2534436	2537285	2537323	2537334

The space groups of compounds **1,2,4** did not change at low temperature and room temperature, and compounds **3** and **5** had phase transitions at low temperature and room temperature. The molecular formula of compound **1** is $C_{32}H_{61}MnN_7O_{12}S_4$ crystallizes in the orthorhombic *P*nma centrosymmetric space group at low temperature and room temperature. The lattice parameters at 100 K are a = 22.8197 (17) Å, b = 14.6192 (9) Å, c = 13.9377 (11) Å, α = 90 °, β = 90 °, γ = 90 °, V = 4649.7 (6) Å^3. With the increase of temperature, the unit cell parameters have no obvious change. The molecular formula of compound **2** is $C_{31}H_{60}MnN_6O_{12}S_4$, which crystallizes in the triclinic *P*-1 centrosymmetric space group. The lattice parameters at 100 K are a = 11.4707 (9) Å, b = 11.6138 (7) Å, c = 17.5560 (14) Å, α = 93.124 (6) °, β = 107.604 (7) °, γ = 92.318 (6) °, V = 2222.0 (3) Å^3. The cell parameters at 293 K are a = 11.7273 (7) Å, b = 11.7655 (5) Å, c = 17.6374 (9) Å, α = 108.552 (4) °, β = 91.421 (5) °, γ = 91.703 (4) °, V = 2304.6 (2) Å^3. With the increase of temperature, the cell parameters *a, b, c, α, β, γ* and *V* all increase, indicating that compound **2** may undergo isostructural phase transfer. The molecular formula of compound **4** is $C_{33}H_{64}MnN_6O_{12}S_4$ and crystallizes in the monoclinic $P2_1$ / n centrosymmetric space group. At 100 K, the cell parameters are a = 13.0612 (8) Å, b = 18.4269 (8) Å, c = 20.2547 (11) Å, α = 90 °, β = 105.641 (6) °, γ = 90 °, V = 4694.3 (5) Å^3. At 293 K, the cell parameters are a = 12.8120 (12) Å, b = 18.6685 (15) Å, c = 21.4317(17) Å, α = 90 °, β = 105.364 (9) °, γ = 90 °, V = 4942.9 (8) Å^3. The molecular formula of compound **3** is $C_{32}H_{62}MnN_6O_{12}S_4$. It crystallizes in the triclinic *P*-1 centrosymmetric space group at low temperature and in the monoclinic *C*2 / c centrosymmetric space group at high temperature. The lattice parameters at 100 K are a = 12.2956 (13) Å, b = 19.4154 (12) Å, c = 21.0682 (12) Å, α = 100.516 (5) °, β = 91.465 (6) °, γ = 107.697 (7) °, V = 4693.3 (7) Å^3. At 293 K, the cell parameters are a = 21.508 (2) Å, b = 12.2104 (12) Å, c = 19.609 (2) Å, α = 90 °, β = 110.368 (12) °, γ = 90 °, V = 1677.0 (3) Å^3. With the increase of temperature, the unit cell parameters *a, b, c, α, β, γ, V* have changed significantly, indicating that compound **3** may undergo isostructural phase transfer. The molecular formula of compound **5** is $C_{34}H_{66}MnN_6O_{12}S_4$. It crystallizes in the monoclinic $P2_1$ / c centrosymmetric space group at low temperature and in the orthorhombic *P*nma centrosymmetric space group at high temperature. The lattice parameters at 100 K are a = 14.3767 (17) Å, b = 13.6354 (9) Å, c = 24.7007 (18) Å, α = 90 °, β = 92.218 (9) °, γ = 90 °, V = 4693.3 (7) Å^3. At 293 K, the cell parameters are a = 24.663 (2) Å, b = 14.4855 (14) Å, c = 14.0493 (13) Å, α = 90 °, β = 90 °, γ = 90 °, V = 5019.2 (8) Å^3. With the increase of temperature, the cell parameters *a, b, c, β, V* have

changed significantly, indicating that compound **5** may undergo isostructural phase transfer.

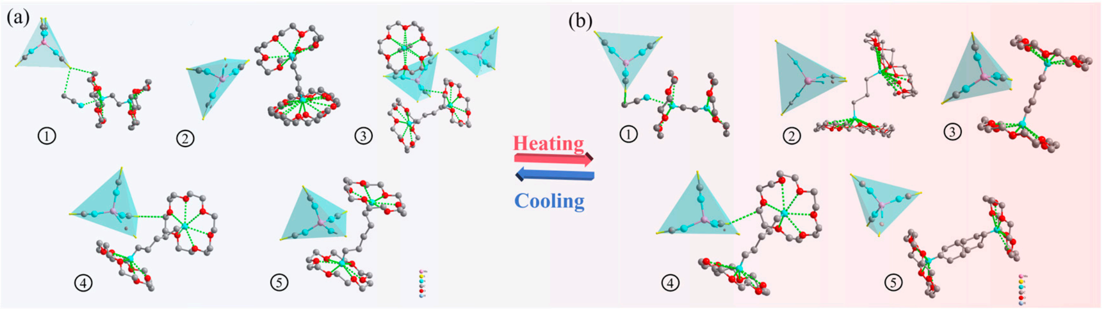

Figure S2 Minimal components of compounds **1–5** at LT (a) and RT (b).

Table S2 The partial bond length (Å) and bond angle (°) of compound **1** at 100 K and 293 K were obtained.

100K			
Mn1-N1	2.049(3)	N1-Mn1-N1	111.23(16)
Mn1-N2	2.056(4)	N1-Mn1-N2	110.46(11)
Mn1-N3	2.065(4)	N1-Mn1-N3	110.17(10)
S1-C19	1.615(5)	N2-Mn1-N3	104.13(16)
S2-C17	1.627(5)	C18-N1-Mn1	167.4(3)
S3-C18	1.626(3)	C19-N2-Mn1	171.3(4)
N1-C18	1.159(4)	C17-N3-Mn1	174.1(4)
N2-C19	1.167(6)	N3-C17-S2	179.2(3)
N3-C17	1.159(6)	N1-C18-S3	179.2(3)
N4-C15	1.133(7)	N2-C19-S1	179.9(4)
C15-C16	1.450(8)	C8-C7-N5	110.7(5)
N5-C7	1.485(6)	C7-C8-N6	111.2(5)
N6-C8	1.495(7)	N4-C15-C16	178.3(6)
C7-C8	1.445(8)		
293K			
Mn1-N1	2.029(6)	N1-Mn1-N1	111.4(3)
Mn1-N2	2.046(9)	N1-Mn1-N2	109.7(2)
Mn1-N3	2.020(8)	N1-Mn1-N3	110.5(19)
S1-C19	1.571(10)	N2-Mn1-N3	104.9(3)
S2-C17	1.588(9)	C18-N1-Mn1	169.3(5)
S3-C18	1.587(6)	C19-N2-Mn1	175.7(7)
N1-C18	1.140(8)	C17-N3-Mn1	174.8(7)
N2-C19	1.117(10)	N3-C17-S2	178.9(7)
N3-C17	1.138(10)	N1-C18-S3	178.6(6)
N4-C15	1.105(17)	N2-C19-S1	179.9(8)
C15-C16	1.381(17)	C8-C7-N5	179.0(3)
N5-C7	1.190(2)	C7-C8-N6	167.0(3)
N6-C8	1.260(2)	N4-C15-C16	176.5(17)

C7-C8	1.284(17)

Table S3 The partial bond length (Å) and bond angle (°) of compound **2** at 100 K and 293 K were obtained

100K			
Mn1-N1	2.136(3)	N1-Mn1-N2	91.23(9)
Mn1-N2	2.114(2)	N1-Mn1-N3	105.67(10)
Mn1-N3	2.075(2)	N1-Mn1-N4	92.18(9)
Mn1-N4	2.104(2)	N2-Mn1-N3	107.89(9)
S1-C19	1.635(3)	N2-Mn1-N4	140.89(10)
S2-C16	1.631(3)	N3-Mn1-N4	108.58(10)
S3-C18	1.640(3)	C19-N1-Mn1	163.7(2)
S4-C17	1.621(3)	C16-N2-Mn1	170.6(2)
N1-C19	1.158(4)	C17-N3-Mn1	169.3(2)
N2-C16	1.160(3)	C18-N4-Mn1	170.8(2)
N3-C17	1.165(4)	N2-C16-S2	179.0(3)
N4-C18	1.159(4)	N3-C17-S4	179.3(3)
N5-C13	1.483(3)	N4-C18-S3	179.6(3)
N6-C15	1.486(3)	N1-C19-S1	179.6(3)
C13-C14	1.506(4)	C14-C13-N5	111.7(2)
C14-C15	1.504(4)	C15-C14-C13	111.5(2)
		N6-C15-C14	111.7(2)
**293K**			
Mn1-N1	2.106(3)	N1-Mn1-N2	91.14(11)
Mn1-N2	2.132(3)	N1-Mn1-N3	139.35(12)
Mn1-N3	2.092(3)	N1-Mn1-N4	109.38(11)
Mn1-N4	2.076(3)	N2-Mn1-N3	91.97(12)
S1-C17	1.622(4)	N2-Mn1-N4	105.68(12)
S2-C16	1.623(4)	N3-Mn1-N4	108.69(13)
S3-C19	1.615(4)	C18-N1-Mn1	172.4(3)
S4-C18	1.614(4)	C17-N2-Mn1	161.5(3)
N1-C18	1.151(4)	C16-N3-Mn1	171.9(3)
N2-C17	1.144(4)	C19-N4-Mn1	171.8(3)
N3-C16	1.146(4)	N3-C16-S2	179.5(3)
N4-C19	1.138(4)	N2-C17-S1	179.9(4)
N5-C13	1.467(4)	N1-C18-S4	179.0(3)
N6-C15	1.468(4)	N4-C19-S3	179.0(3)
C13-C14	1.460(4)	C14-C13-N5	115.2(3)
C14-C15	1.479(4)	C15-C14-C13	115.0(3)
		N6-C15-C14	114.4(3)

Table S4 The partial bond length (Å) and bond angle (°) of compound **3** at 100 K and 293 K were

obtained.

100K			
Mn1-N1	2.063(10)	N1-Mn1-N2	109.0(4)
Mn1-N2	2.052(10)	N1-Mn1-N3	113.5(4)
Mn1-N3	2.054(10)	N1-Mn1-N4	105.5(4)
Mn1-N4	2.043(9)	N2-Mn1-N3	101.7(4)
S1-C63	1.632(12)	N2-Mn1-N4	118.1(4)
S2-C62	1.617(13)	N3-Mn1-N4	109.3(4)
S3-C61	1.615(11)	C63-N1-Mn1	162.9(10)
S4-C64	1.630(13)	C62-N2-Mn1	161.1(10)
N1-C63	1.140(14)	C61-N3-Mn1	165.5(10)
N2-C62	1.158(15)	C64-N4-Mn1	172.0(9)
N3-C61	1.160(14)	N3-C61-S3	179.7(13)
N4-C64	1.154(14)	N2-C62-S2	178.6(13)
Mn2-N7	2.062(10)	N1-C63-S1	179.5(13)
Mn2-N8	2.060(1)	N4-C64-S4	178.6(10)
Mn2-N9	2.062(11)	N7-Mn2-N8	109.0(4)
Mn2-N10	2.060(1)	N7-Mn2-N9	112.9(5)
S5-C29	1.629(12)	N7-Mn2-N10	108.3(4)
S6-C30	1.607(14)	N8-Mn2-N9	104.3(4)
S7-C32	1.606(12)	N8-Mn2-N10	116.4(4)
S8-C31	1.615(14)	N9-Mn2-N10	106.0(4)
N7-C29	1.136(15)	C29-N7-Mn2	168.1(10)
N8-C30	1.161(15)	C30-N8-Mn2	164.6(10)
N9-C32	1.1680(14)	C32-N9-Mn2	163.4(10)
N10-C31	1.1550(15)	C31-N10-Mn2	170.2(10)
N5-C13	1.4680(13)	N7-C29-S5	179.0(12)
C13-C14	1.5360(13)	N8-C30-S6	178.0(12)
C14-C15	1.4940(15)	N10-C31-S8	178.2(11)
C15-C16	1.5330(13)	N9-C32-S7	179.0(12)
C16-N6	1.4900(13)	C14-C13-N5	111.5(8)
N11-C48	1.4930(13)	C15-C14-C13	111.5(9)
C48-C47	1.4900(14)	C16-C15-C14	111.0(9)
C47-C46	1.5240(15)	N6-C16-C15	111.0(8)
C46-C45	1.4930(15)	C47-C48-N11	113.0(9)
C45-N12	1.4860(14)	C46-C47-C48	112.2(9)
		C45-C46-C47	112.2(9)
		N12-C45-C46	112.4(9)
293K			
Mn1-N2	2.038(3)	N2-Mn1-N2	102.5(2)
Mn1-N3	2.029(4)	N2-Mn1-N3	115.49(14)

S1-C16	1.616(4)	N2-Mn1-N3	108.24(14)
S2-C15	1.605(4)	N3-Mn1-N3	107.1(2)
N2-C15	1.133(5)	C15-N2-Mn1	165.2(3)
N3-C16	1.137(5)	C16-N3-Mn1	169.2(3)
N1-C13	1.465(5)	N2-C15-S2	179.1(5)
C13-C14	1.420(6)	N3-C16-S3	179.4(4)
C14-C14	1.454(7)	N1-C13-C14	117.0(3)
		C13-C14-C14	117.5(4)

Table S5 The partial bond length (Å) and bond angle (°) of compound **4** at 100 K and 293 K were obtained.

100K			
Mn1-N3	2.064(3)	N4-Mn1-N3	110.14(13)
Mn1-N4	2.041(4)	N4-Mn1-N5	109.30(14)
Mn1-N5	2.049(3)	N4-Mn1-N6	113.56(13)
Mn1-N6	2.047(3)	N5-Mn1-N3	108.66(12)
S1-C31	1.610(4)	N5-Mn1-N6	109.79(12)
S2-C30	1.620(4)	N6-Mn1-N3	105.24(13)
S3-C32	1.613(4)	C32-N3-Mn1	166.1(3)
S4-C33	1.610(5)	C33-N4-Mn1	164.4(3)
N3-C32	1.155(5)	C30-N5-Mn1	174.4(3)
N4-C33	1.163(5)	C31-N6-Mn1	169.3(3)
N5-C30	1.165(5)	N5-C30-S2	179.0(4)
N6-C31	1.178(5)	N6-C31-S1	178.6(3)
N1-C13	1.495(4)	N3-C32-S3	178.8(4)
N2-C17	1.498(4)	N4-C33-S4	179.0(4)
C13-C14	1.509(5)	N1-C13-C14	112.1(3)
C14-C15	1.515(5)	C13-C14-C15	111.2(3)
C15-C16	1.522(5)	C14-C15-C16	114.7(3)
C16-C17	1.510(5)	C15-C16-C17	113.7(3)
		C16-C17-N2	110.0(3)
293K			
Mn1-N3	1.997(11)	N3-Mn1-N4	108.2(5)
Mn1-N4	2.140(2)	N3-Mn1-N5	110.6(5)
Mn1-N5	1.998(15)	N3-Mn1-N6	103.5(5)
Mn1-N6	2.004(14)	N4-Mn1-N5	104.2(5)
S1-C31	1.661(18)	N4-Mn1-N6	118.2(6)
S2-C30	1.542(17)	N5-Mn1-N6	112.1(5)
S3-C32	1.548(14)	C32-N3-Mn1	167.6(12)
S4-C33	1.502(14)	C33-N4-Mn1	162.4(13)
N3-C32	1.067(13)	C30-N5-Mn1	175.1(15)
N4-C33	1.180(2)	C31-N6-Mn1	166.7(15)

N5-C30	1.145(17)	N5-C30-S2	176.3(17)
N6-C31	1.082(17)	N6-C31-S1	176.1(15)
N1-C13	1.409(14)	N3-C32-S3	176.5(13)
N2-C17	1.518(2)	N4-C33-S4	177.7(17)
C13-C14	1.449(15)	N1-C13-C14	112.4(10)
C14-C15	1.411(2)	C13-C14-C15	112.9(13)
C15-C16	1.639(2)	C14-C15-C16	100.2(14)
C16-C17	1.162(3)	C15-C16-C17	114.0(18)
		C16-C17-N2	120.9(17)

Table S6 The partial bond length (Å) and bond angle (°) of compound **5** at 100 K and 293 K were obtained.

100K			
Mn1-N1	2.067(8)	N1-Mn1-N2	110.2(3)
Mn1-N2	2.023(8)	N1-Mn1-N3	110.3(4)
Mn1-N3	2.063(9)	N1-Mn1-N4	110.4(4)
Mn1-N4	2.049(8)	N2-Mn1-N3	107.1(4)
S1-C3	1.625(10)	N2-Mn1-N4	112.8(4)
S2-C2	1.634(10)	N3-Mn1-N4	105.9(3)
S3-C4	1.627(10)	C1-N1-Mn1	168.7(9)
S4-C1	1.620(9)	C4-N2-Mn1	164.5(8)
N1-C1	1.133(12)	C2-N3-Mn1	171.0(10)
N2-C4	1.193(12)	C3-N4-Mn1	164.1(8)
N3-C2	1.135(13)	N1-C1-S4	177.9(9)
N4-C3	1.152(12)	N3-C2-S2	176.6(11)
N5-C29	1.520(10)	N4-C3-S1	179.5(10)
N6-C34	1.496(10)	N2-C4-S3	177.4(9)
C29-C30	1.505(12)	C30-C29-N5	109.3(6)
C30-C31	1.559(11)	C31-C30-C29	111.8(7)
C31-C32	1.492(12)	C32-C31-C30	111.3(7)
C32-C33	1.552(13)	C33-C32-C31	111.8(8)
C33-C34	1.484(12)	C34-C33-C32	113.1(8)
		N6-C34-C33	112.0(7)
293K			
Mn1-N1	2.076(8)	N1-Mn1-N2	107.1(3)
Mn1-N2	2.020(8)	N1-Mn1-N3	108.4(2)
Mn1-N3	2.017(6)	N2-Mn1-N3	111.3(2)
S1-C1	1.614(7)	N3-Mn1-N3	110.2(2)
S2-C2	1.588(10)	C3-N1-Mn1	176.1(13)
S3-C3	1.546(12)	C2-N2-Mn1	170.8(8)
N1-C3	1.095(12)	C1-N3-Mn1	170.4(6)

N2-C2	1.145(9)	N3-C1-S1	178.0(6)
N3-C1	1.153(7)	N2-C2-S2	178.3(8)
N4-C10	1.503(7)	N1-C3-S3	178.8(19)
N5-C15	1.480(9)	N4-C10-C11	111.4(5)
C10-C11	1.491(10)	C10-C11-C12	112.6(8)
C11-C12	1.536(12)	C11-C12-C13	115.0(8)
C12-C13	1.447(13)	C12-C13-C14	116.7(10)
C13-C14	1.523(14)	C13-C14-C15	125.7(15)
C14-C15	1.205(15)	C14-C15-N5	126.8(10)

The partial bond lengths and bond angles of compounds **1-5** are listed in Tables S2-S6. The data in the table show that at 100 K, the Mn-N bond length range of inorganic anions in compounds **1-5** is 2.023-2.114 Å, S-C bond length range is 1.613-1.634 Å, N-C bond length range is 1.133-1.193 Å, N-C bond length range in organic cations is 1.483-1.520 Å, C-C bond length range is 1.445-1.559 Å. The N-Mn-N bond angle ranges from 91.23 ° to 113.50 °. At 293 K, the Mn-N bond length range of inorganic anions in compounds 1-5 is 2.020-2.143 Å, the S-C bond length range is 1.542-1.661 Å, and the N-C bond length range is 1.095-1.153 Å. The N-C bond length range of organic cations is 1.19-1.516 Å, and the C-C bond length range is 1.205-1.639 Å. The N-Mn-N bond angle ranges from 91.14 ° to 139.35 °.

Table S7 The distance between two N atoms in diamine ions in compounds **1-5**

	**1**	**2**	**3**	**4**	**5**
100 K	3.745 Å	4.942 Å	6.269 Å	6.867 Å	8.011 Å
293 K	3.706 Å	4.947 Å	6.217 Å	7.026 Å	8.134 Å
differentials	-0.039 Å	0.005 Å	-0.052 Å	0.159 Å	0.123 Å

Table S7 shows the distance between two N atoms in diamine ions of compounds **1-5**. The results show that the distance between two N atoms in compound **1** is 3.745 Å at 100 K and 3.706 Å at 293 K, which is reduced by 0.039 Å. The distance between two N atoms in compound **2** is 4.942 Å at 100 K and 4.947 Å at 293 K, which is increased by 0.005 Å. Compound **3** contains two components of cations at 100 K. The distance between the two N atoms was measured to be 6.256 Å and 6.281 Å, respectively. The average distance was 6.269 Å, and it was 6.217 Å at 293 K, which

was reduced by 0.052 Å. In compound **4**, the distance between two N atoms is 6.867 Å at 100 K and 7.026 Å at 293 K, which increases by 0.159 Å. In compound **5**, the distance between two N atoms is 8.011 Å at 100 K and 8.134 Å at 293 K, which increases by 0.123 Å. It can be seen that the diamine chain of compound **1** and compound **3** is longer at 100 K, and the diamine chain of the remaining compounds increases with the increase of temperature.

Table S8 Some hydrogen bond parameters of compound **1** at 100K and 293K

D-H·A	d(D·H) Å	d(H·A) Å	d(D·A) Å	D-H·A(°)
		100 K		
N5-H5···O1	0.8902(33)	1.9613(29)	2.8205(46)	161.754(70)
N5-H5···O2	0.8901(18)	2.5137(21)	2.9045(27)	107.229(47)
N5-H5···O3	0.8901(18)	1.9620(21)	2.8488(29)	174.072(62)
N5-H5···O4	0.8901(23)	2.5287(29)	2.9692(47)	111.218(167)
N6-H6···O5	0.8904(40)	2.1309(32)	2.9054(50)	144.992(52)
N6-H6···O6	0.8904(40)	2.5162(22)	2.9926(30)	108.760(53)
N6-H6···O7	0.8901(19)	1.9555(23)	2.8411(31)	173.004(67)
N6-H6···O8	0.8901(26)	2.5508(27)	2.8893(50)	104.109(22)
N5-H5···N4	0.8901(23)	2.6910(54)	2.8663(64)	92.172(9)
C1-H1···S2	0.9697(31)	2.9812(12)	3.5133(31)	115.795(167)
C1-H1···S1	0.9705(28)	2.9187(13)	3.7808(31)	148.559(166)
C16-H16···S3	0.9602(18)	3.1138(9)	3.8434(18)	134.002(16)
		293 K		
N5-H5···O1	0.8903(62)	1.9955(47)	2.8009(82)	149.777(20)
N5-H5···O2	0.8901(21)	2.2564(34)	2.9117(47)	130.233(77)
N5-H5···O3	0.8899(55)	2.0328(44)	2.8325(57)	148.923(124)
N5-H5···O4	0.8899(55)	2.2392(45)	2.8444(82)	124.979(22)
N6-H6···O5	0.8908(66)	2.0144(68)	2.8668(97)	159.764(26)
N6-H6···O6	0.8908(66)	2.3360(6)	2.9059(72)	121.812(160)
N6-H6···O7	0.8901(24)	1.9964(48)	2.8559(59)	161.923(132)
N6-H6···O8	0.8898(48)	2.2899(58)	2.8467(86)	120.498(26)
N5-H5···N4	0.8899(55)	2.5962(137)	2.8721(155)	98.922(16)
C1-H1···S2	0.9691(65)	3.0472(22)	3.6193(61)	119.123(337)
C1-H1···S1	0.9705(56)	2.8898(26)	3.7326(63)	145.800(333)
C16-H16···S3	0.9599(47)	3.1205(19)	3.9044(53)	139.931(35)

Table S9 Some hydrogen bond parameters of compound **2** at 100K and 293K

D-H·A	d(D·H) Å	d(H·A) Å	d(D·A) Å	D-H·A(°)
		100 K		
N5-H22···O6	0.8904(25)	2.0023(22)	2.8838(33)	170.258(160)
N5-H22···O5	0.8904(25)	2.5385(20)	2.9921(30)	112.308(152)

N5-H23···O4	0.8898(25)	1.9993(21)	2.8888(32)	178.145(154)
N5-H23···O3	0.8898(25)	2.5586(21)	2.9109(33)	104.436(150)
N5-H24···O2	0.8901(21)	2.0311(19)	2.9166(28)	173.017(153)
N5-H24···O1	0.8901(21)	2.5312(23)	2.8619(33)	102.704(156)
N6-H27···O16	0.8900(25)	2.3894(46)	2.9774(52)	123.760(185)
N6-H27···O12	0.8900(25)	2.0297(37)	2.9085(45)	169.098(184)
N6-H27···O17	0.8900(25)	2.8354(70)	3.6837(74)	159.847(197)
N6-H27···O11	0.8900(25)	2.4746(42)	2.8922(51)	109.203(174)
N6-H25···O18	0.8900(2)	2.3922(45)	2.8824(48)	114.912(163)
N6-H25···O10	0.8900(2)	2.0156(46)	2.8599(49)	157.895(198)
N6-H25···O13	0.8900(2)	1.9852(42)	2.8207(45)	155.794(184)
N6-H25···O9	0.8900(2)	2.5013(34)	3.0286(38)	118.493(171)
N6-H26···O14	0.8900(23)	3.0748(58)	3.6074(64)	120.373(187)
N6-H26···O8	0.8900(23)	1.9912(47)	2.8729(52)	170.635(190)
N6-H26···O15	0.8900(23)	2.0133(46)	2.8778(51)	163.535(186)
N6-H26···O7	0.8900(23)	2.4905(40)	2.9532(43)	112.922(160)
C2-HC···S1	0.9699(28)	2.8841(8)	3.7549(29)	149.894(183)
C26-H40B···S1	1.1298(61)	3.0225(8)	3.5840(71)	110.872(357)
C11-HT···S3	0.9699(28)	3.1042(9)	3.5286(35)	108.135(181)
C7-HL···S2	0.9700(37)	2.7570(7)	3.5453(36)	138.852(201)
C24-H6···S2	0.9708(76)	2.8669(7)	3.8053(80)	169.832(489)
C10-H10···S2	0.9701(37)	3.2166(8)	3.8323(37)	123.006(210)
		293 K		
N6-H6D···O7	0.8895(23)	2.3506(46)	2.9228(52)	122.167(185)
N6-H6D···O7A	0.8895(23)	2.3419(322)	3.1172(332)	145.687(795)
N6-H6D···O8	0.8895(23)	2.1915(34)	3.0096(41)	152.692(178)
N6-H6D···O8A	0.8895(23)	2.1602(245)	2.8845(242)	138.156(643)
N6-H6E···O9	0.8904(27)	2.3964(38)	2.9312(48)	118.809(179)
N6-H6E···O9A	0.8904(27)	2.1923(252)	2.9639(257)	144.699(645)
N6-H6E···O10	0.8904(27)	2.0121(38)	2.8666(46)	160.440(188)
N6-H6E···O10A	0.8904(27)	2.3134(231)	2.9880(238)	132.514(605)
N6-H6C···O11	0.8902(25)	2.3372(45)	2.9502(48)	126.046(179)
N6-H6C···O11A	0.8902(25)	2.2876(241)	3.0636(254)	145.586(668)
N6-H6C···O12	0.8902(25)	2.0767(44)	2.8986(50)	153.079(183)
N6-H6C···O12A	0.8902(25)	2.1824(331)	2.8582(323)	132.281(706)
N5-H5C···O1	0.8899(24)	2.0559(65)	2.8733(69)	152.223(227)
N5-H5C···O1A	0.8899(24)	2.0260(73)	2.9115(77)	173.183(250)
N5-H5C···O2	0.8899(24)	2.2287(59)	2.9306(63)	128.813(212)
N5-H5C···O2A	0.8899(24)	2.5410(73)	2.9642(75)	109.882(221)
N5-H5E···O3	0.8901(27)	2.0733(74)	2.9144(80)	157.201(258)
N5-H5E···O3A	0.8901(27)	2.0214(78)	2.9096(83)	175.485(262)
N5-H5E···O4	0.8901(27)	2.3024(70)	2.8882(78)	123.258(221)
N5-H5E···O4A	0.8901(27)	2.5044(70)	2.8719(79)	105.398(224)
N5-H5D···O5	0.8900(2)	2.0321(64)	2.8477(65)	151.798(242)

N5-H5D···O5A	0.8900(2)	1.9392(64)	2.8002(66)	162.297(258)
N5-H5D···O6	0.8900(2)	2.3778(69)	3.0053(68)	127.648(233)
N5-H5D···O6A	0.8900(2)	2.5636(72)	2.9674(72)	108.417(223)
C8A-H12A···S1	1.1181(85)	3.0971(14)	3.6828(96)	113.134(487)
C24A-H24D···S2	0.9658(339)	2.7290(11)	3.4975(273)	136.955(1420)
C31-H31A···S4	0.9701(77)	2.8008(10)	3.5528(71)	134.916(410)
C28-H28B···S4	0.9707(67)	3.3824(11)	3.9309(56)	117.868(310)
C18A-H18A···S4	0.9703(96)	2.9816(10)	3.9501(98)	176.041(578)

Table S10 Some hydrogen bond parameters of compound **3** at 100K and 293K

D-H·A	d(D·H) Å	d(H·A) Å	d(D·A) Å	D-H·A(°)
		100 K		
N5-H5···O1	0.8905(83)	2.6121(85)	2.9969(123)	107.062(605)
N5-H5···O2	0.8905(83)	1.9942(72)	2.8805(111)	173.257(640)
N5-H5···O3	0.8898(106)	2.5821(68)	3.0031(113)	109.843(610)
N5-H5···O4	0.8898(106)	2.0150(88)	2.8996(138)	172.613(634)
N5-H5···O5	0.8903(86)	2.5090(85)	2.9756(128)	113.294(603)
N5-H5···O6	0.8903(86)	2.0324(72)	2.8798(112)	158.619(586)
N6-H6···O7	0.8907(105)	2.0052(88)	2.8746(138)	164.918(634)
N6-H6···O8	0.8897(86)	2.4329(85)	2.9812(128)	120.191(603)
N6-H6···O9	0.8897(86)	2.0529(80)	2.8867(119)	155.622(598)
N6-H6···O10	0.8894(84)	2.5158(84)	2.9759(123)	112.816(608)
N6-H6···O11	0.8894(84)	1.9983(86)	2.8734(121)	167.659(660)
N6-H6···O12	0.8907(105)	2.5421(80)	3.0216(121)	114.453(620)
C22-H22···S6	0.9697(106)	3.0812(38)	3.4834(127)	106.526(671)
C23-H23···S1	0.9698(126)	2.8668(29)	3.5907(130)	132.188(746)
C6-H6···S1	0.9704(118)	2.8914(34)	3.6888(140)	140.112(739)
C34-H34···S4	0.9700(158)	3.0730(35)	3.6795(153)	121.941(868)
C35-H35···S7	0.9700(103)	3.4189(35)	3.7181(124)	100.373(656)
C12-H12···S2	0.9692(106)	2.9708(35)	3.3817(122)	106.868(660)
C28-H28···S5	0.9703(117)	2.9891(38)	3.7277(146)	133.862(746)
C1-H1···S5	0.9699(123)	2.8519(34)	3.5389(118)	128.584(659)
C57-H57···S3	0.9699(116)	3.0347(35)	3.6538(131)	122.935(741)
C56-H56···S8	0.9703(119)	3.3824(36)	3.7211(127)	102.890(72)
C50-H50···S7	0.9698(117)	3.0642(33)	3.7578(124)	129.637(824)
		293 K		
N1-H1···O1	0.8901(33)	2.4935(36)	2.9785(48)	114.797(198)
N1-H1···O2	0.8901(26)	1.9631(37)	2.8522(45)	176.695(213)
N1-H1···O3	0.8901(26)	2.5979(43)	2.9879(49)	107.438(197)
N1-H1···O4	0.8896(25)	2.0507(37)	2.8914(45)	157.184(189)
N1-H1···O5	0.8896(25)	2.4908(38)	2.9848(47)	115.581(188)
N1-H1···O6	0.8901(33)	2.0792(44)	2.9543(55)	167.406(212)
C3-H3···S1	0.9704(60)	3.2003(17)	3.9038(76)	130.773(394)

C10-H10···S2	0.9689(66)	0.0330(15)	3.7489(54)	131.790(299)
C8-H8···S1	0.9692(90)	3.0316(14)	3.7600(98)	133.004(571)

Table S11 Some hydrogen bond parameters of compound **4** at 100K and 293K

D-H·A	d(D·H) Å	d(H·A) Å	d(D·A) Å	D-H·A(º)
		100 K		
N1-H1···O1	0.8896(28)	2.0690(25)	2.9144(37)	158.325(183)
N1-H1···O2	0.8897(26)	2.5910(27)	2.9614(38)	105.926(185)
N1-H1···O3	0.8897(26)	1.9791(23)	2.8650(35)	173.636(181)
N1-H1···O4	0.8904(23)	2.5007(22)	2.9902(33)	115.180(179)
N1-H1···O5	0.8904(23)	2.0010(23)	2.8844(33)	171.356(191)
N1-H1···O6	0.8896(28)	2.5263(29)	2.9565(37)	110.408(181)
N2-H2···O7	0.8894(24)	2.6402(25)	3.0481(38)	109.000(179)
N2-H2···O8	0.8894(24)	2.0049(23)	2.8902(34)	173.386(186)
N2-H2···O9	0.8903(25)	2.5223(23)	2.9419(34)	109.510(166)
N2-H2···O10	0.8903(25)	1.9808(25)	2.8584(36)	168.328(178)
N2-H2···O11	0.8900(26)	2.6145(22)	3.0259(31)	109.172(173)
N2-H2···O12	0.8900(26)	1.9181(22)	2.8025(34)	172.219(174)
C10-H10···S3	0.9707(40)	3.0754(14)	3.6006(47)	115.432(236)
C14-H14···S2	0.9703(33)	3.0932(9)	3.8518(35)	136.128(206)
C24-H24···S4	0.9700(38)	3.0166(11)	3.6886(35)	127.534(203)
		293 K		
N1-H1···O1	0.8906(93)	2.1634(179)	2.9645(206)	149.328(764)
N1-H1···O2	0.8897(92)	2.2911(200)	2.8619(219)	121.827(788)
N1-H1···O3	0.8897(92)	2.0286(184)	2.8861(206)	161.491(794)
N1-H1···O4	0.8898(80)	2.4677(181)	3.0764(196)	126.028(704)
N1-H1···O5	0.8898(80)	2.0559(187)	2.8645(203)	150.581(793)
N1-H1···O6	0.8906(93)	2.4197(160)	3.1894(186)	144.860(689)
C10-H10···S3	0.9697(237)	2.9779(48)	3.7724(263)	139.97(133)
C29-H29···S1	0.9706(248)	2.9080(63)	3.8395(257)	161.174(1424)
C19-H19···S4	0.9712(238)	3.0925(55)	3.4047(234)	100.405(1341)

Table S12 Some hydrogen bond parameters of compound **5** at 100K and 293K

D-H·A	d(D·H) Å	d(H·A) Å	d(D·A) Å	D-H·A(º)
		100 K		
N5-H5···O1	0.9103(58)	1.9734(51)	2.7923(74)	148.815(360)
N5-H5···O2	0.9095(69)	2.3336(57)	2.9458(91)	124.508(456)
N5-H5···O3	0.9095(69)	2.0074(54)	2.8497(85)	153.365(429)
N5-H5···O4	0.9101(61)	2.3215(49)	2.9468(70)	125.698(337)
N5-H5···O5	0.9101(61)	2.0466(57)	2.8889(88)	153.312(418)
N5-H5···O6	0.9103(58)	2.3035(58)	2.9036(89)	123.197(428)
N6-H6···O7	0.9099(81)	2.0159(71)	2.8908(108)	160.808(551)
N6-H6···O8	0.9094(83)	2.4281(73)	2.8191(105)	106.098(497)

N6-H6···O9	0.9094(83)	1.9411(73)	2.8474(111)	174.241(556)
N6-H6···O10	0.9107(73)	2.4935(71)	2.9972(108)	113.581(534)
N6-H6···O11	0.9107(73)	1.9715(51)	2.8658(89)	166.814(481)
N6-H6···O12	0.9099(81)	2.5268(74)	2.9663(112)	110.181(545)
C24-H24···S4	0.9895(123)	2.9930(27)	3.9949(126)	161.744(712)
C21-H31···S1	0.9894(101)	2.9495(23)	3.9130(104)	164.846(588)
C13-H13···S1	0.9890(74)	2.9580(24)	3.4966(82)	115.273(455)
C30-H30···S2	0.9904(97)	3.1118(34)	3.6763(99)	117.546(549)
C32-H32···S3	0.9899(98)	3.2599(25)	3.9532(96)	128.535(530)
		293 K		
N4-H4···O1	0.8900(35)	2.2965(34)	2.9334(56)	128.389(26)
N4-H4···O2	0.8900(35)	2.0731(29)	2.8852(37)	151.206(81)
N4-H4···O3	0.8899(16)	2.2925(27)	2.9169(36)	127.092(66)
N4-H4···O4	0.8896(41)	2.0096(36)	2.8087(57)	148.811(36)
N5-H5···O5	0.8889(43)	2.1521(48)	2.8392(70)	133.624(29)
N5-H5···O6	0.8889(43)	2.1181(44)	2.8716(52)	142.060(121)
N5-H5···O7	0.8897(14)	2.2366(46)	2.9464(51)	136.509(112)
N5-H5···O8	0.8906(42)	2.1419(51)	2.9013(70)	142.751(26)
C13-H13···S1	0.9705(164)	3.0778(16)	3.9625(167)	152.247(923)
C4-H4···S2	0.9701(44)	3.1188(22)	3.6556(50)	116.459(259)
C11-H11···S3	0.9705(99)	3.2136(48)	3.8905(103)	128.309(542)

The partial hydrogen bond lengths and bond angles of compounds **1-5** are listed in Tables S8-S12.The data in the table show that the main hydrogen bond types in compounds **1-5** are N-H···O and C-H···S. At 100 K, the bond length range of N-H···O hydrogen bond in compound **1** is 2.8205-2.9926 Å, and the bond angle range is 91.172-173.004 °. The bond length range of C-H···S hydrogen bond is 3.5133-3.8434 Å, and the bond angle range is 115.795-148.559 °. The bond length range of N-H···O hydrogen bond in compound **2** is 2.8599-3.6074 Å, and the bond angle range is 102.704-173.017 °. The C-H···S hydrogen bond lengths range from 3.5286 to 3.8323 Å. The bond length range of N-H···O hydrogen bond in compound **3** is 2.8734-3.0216 Å, and the bond angle range is 107.062-173.257 °. The bond length range of C-H···S hydrogen bond is 3.3817-3.7578 Å, and the bond angle range is 100.373-140.112 °. The bond length range of N-H···O hydrogen bond in compound **4** is 2.8025-3.0259 Å, and the bond angle range is 105.926-173.386 °. The bond length range of C-H···S hydrogen bond is 3.6006-3.8518 Å. The bond angle range is 115.432-127.534 °. The bond length range of N-H···O hydrogen bond in compound **5** is 2.8191-2.9972 Å, and the bond angle range is 106.098-174.241 °. The bond length range of C-H···S hydrogen bond is 3.4966-3.9949 Å, and the bond angle range is 115.273-164.846 °. At 293 K, the bond length range of N-

H···O hydrogen bond in compound **1** is 2.8009-2.9117 Å, and the bond angle range is 98.922-161.923 °. The bond length range of C-H···S hydrogen bond is 3.6193-3.9044 Å, and the bond angle range is 119.123-145.800 °. The bond length range of N-H···O hydrogen bond in compound **2** is 2.8477-3.1172 Å, and the bond angle range is 105.398-173.183 °. The bond length range of C-H···S hydrogen bond is 3.4975-3.9501 Å, and the bond angle range is 113.134-176.041 °. The bond length range of N-H···O hydrogen bond in compound **3** is 2.8522-2.9848 Å, and the bond angle range is 107.438-176.695 °. The bond length range of C-H···S hydrogen bond is 3.7600-3.9038 Å, and the bond angle range is 130.773-133.004 °. The bond length range of N-H···O hydrogen bond in compound **4** is 2.8619-3.1894 Å, and the bond angle range is 121.827-161.491 °. The bond length range of C-H···S hydrogen bond is 3.4047-3.8395 Å, and the bond angle range is 100.405-161.174 °.The bond length range of N-H···O hydrogen bond of compound **5** is 2.8392-2.9464 Å, and the bond angle range is 127.092-151.206 °.The bond length range of C-H···S hydrogen bond is 3.6556-3.9625 Å, and the bond angle range is 116.459-152.247 °.

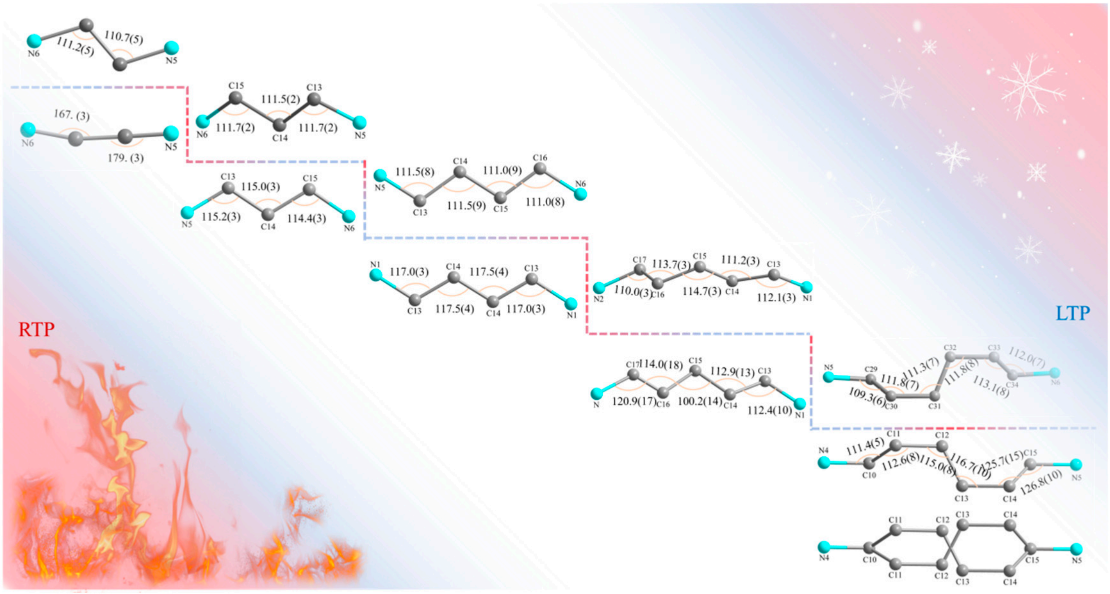

Figure S3 Diamine diagrams of compounds **1-5** at LT and RT

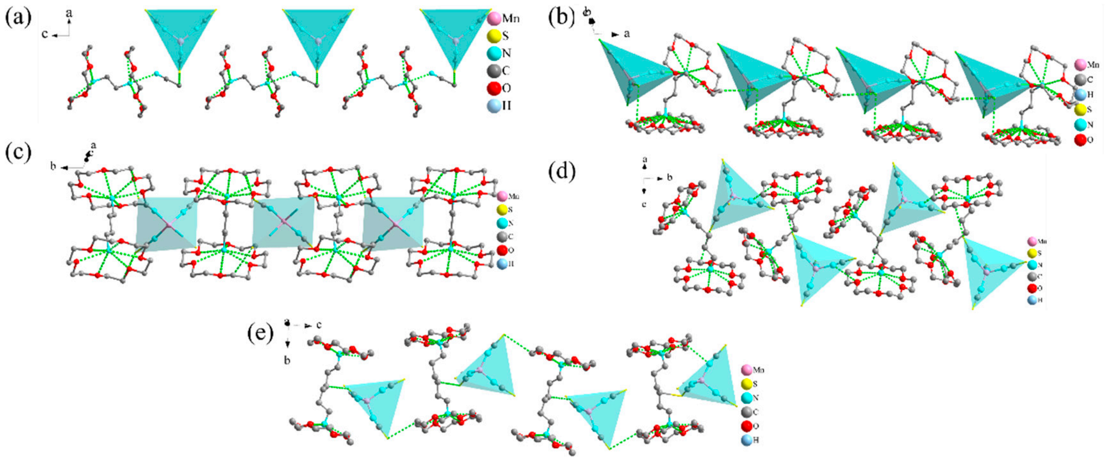

Figure S4 One-dimensional chain diagrams of compounds **1-5** at LT (a) (b) (c) (d) (e)

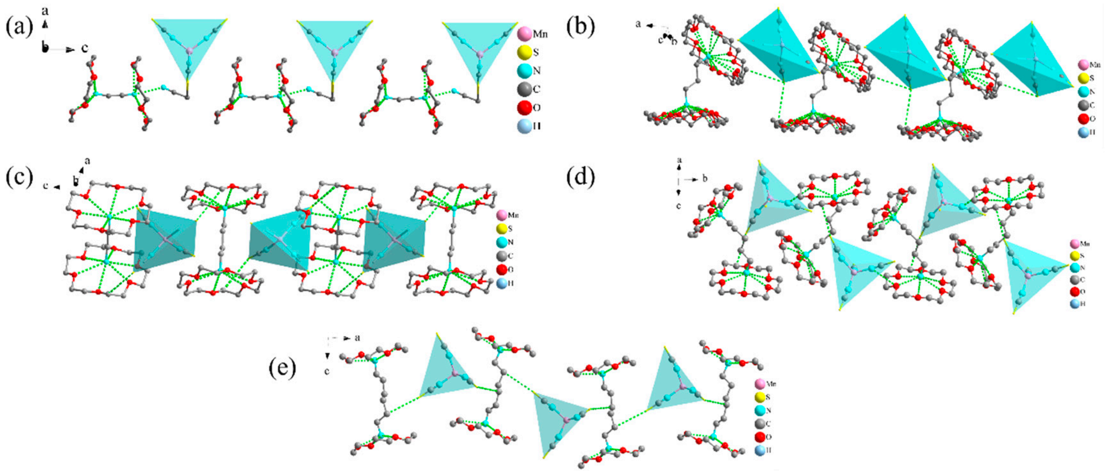

Figure S5 One-dimensional chain diagrams of compounds **1-5** at RT (a) (b) (c) (d) (e)

Figures S4 and S5 are the hydrogen bond chain diagrams of compounds **1-5** at LT and RT. Compounds **1-5** are connected to each other by C-H···S hydrogen bond force. The hydrogen bond chain is composed of a thiocyanato manganese complex and a two-sided diamine crown ether organic cation to form an alternating sequence, thereby forming a one-dimensional infinite chain hydrogen bond diagram.

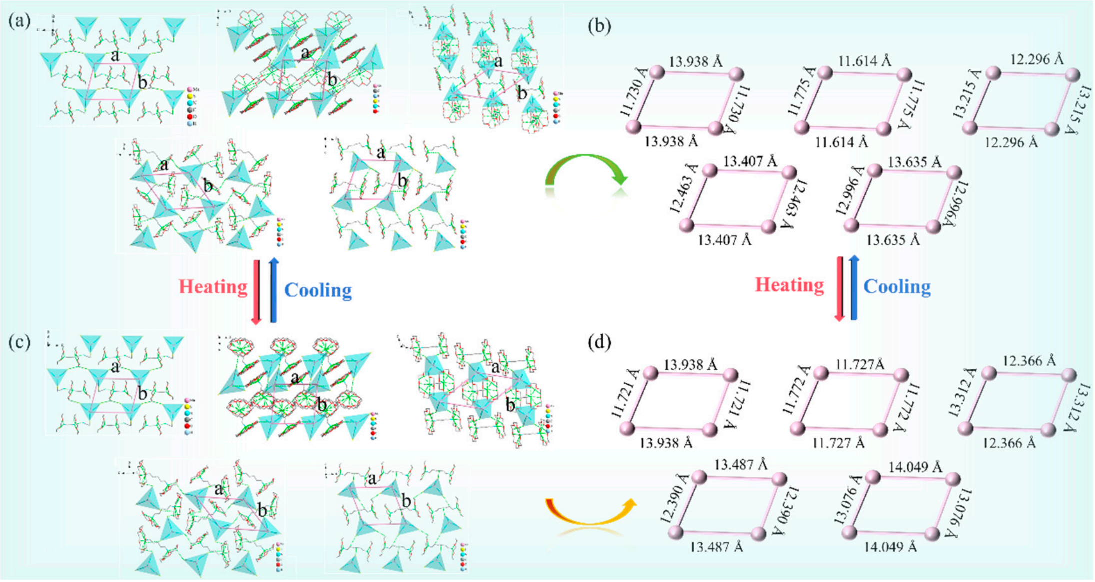

Figure S6 2D hydrogen-bonding networks of compounds **1–5** at LT (a) and RT (c); side lengths of quadrilaterals constructed with manganese atoms as vertices. The longer sides are designated as "a" and the shorter sides as "b". The plot presents the measured a and b values for compound **1–5** at LT (b) and RT (d)

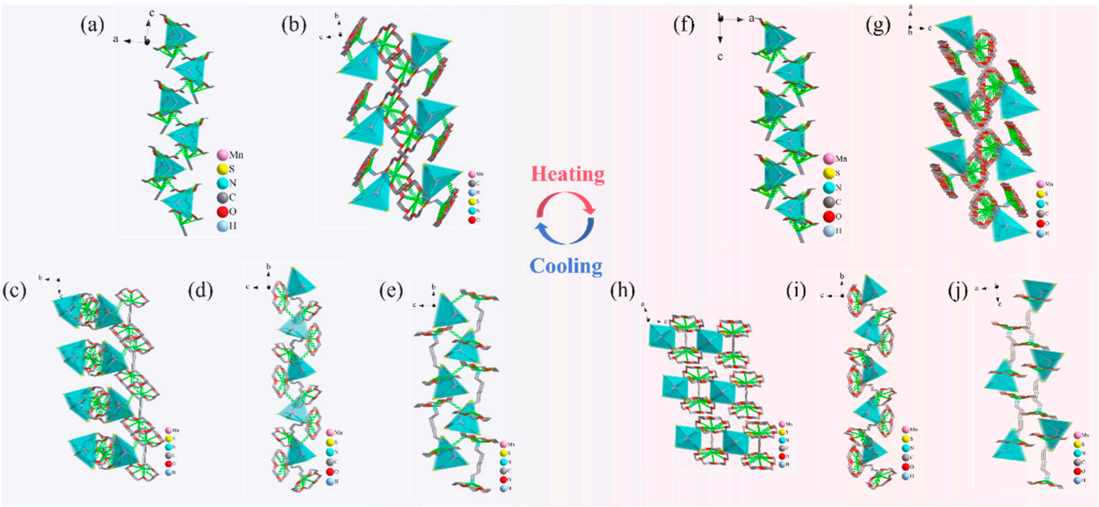

Figure S7 Stacking diagrams of compounds **1-5** at LT (a), (b), (c), (d), and (e), and at RT (f), (g), (h), (i), and (j). The blue polyhedron represents the inorganic anion $[Mn(NCS)_4]^{2-}$ unit, while the organic diamine–crown ether cationic framework is depicted as a wireframe model, clearly illustrating the changes in supramolecular assembly and hydrogen-bond networks at different temperatures.

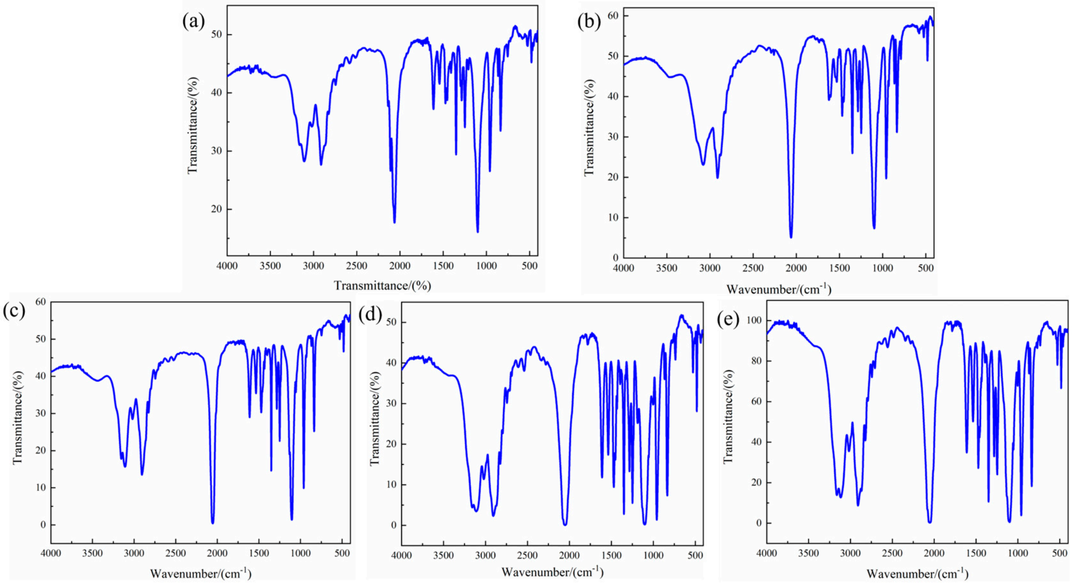

Figure S8 IR spectra of compounds **1-5** (a) (b) (c) (d) (e)

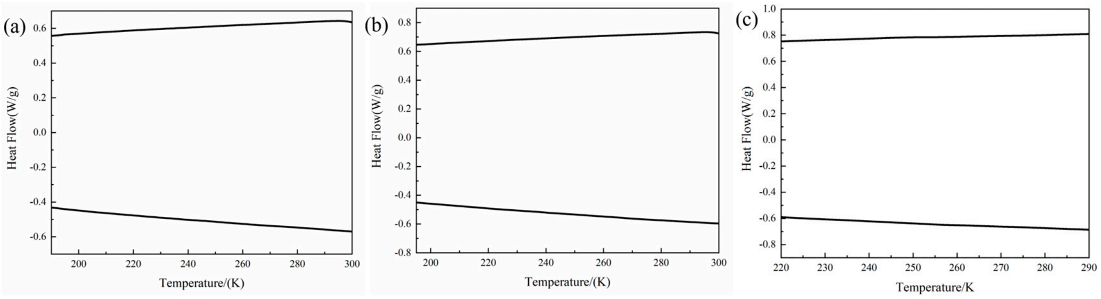

Figure S9 DSC diagram of compounds **1**、**2**、**4** (a) (b) (c)

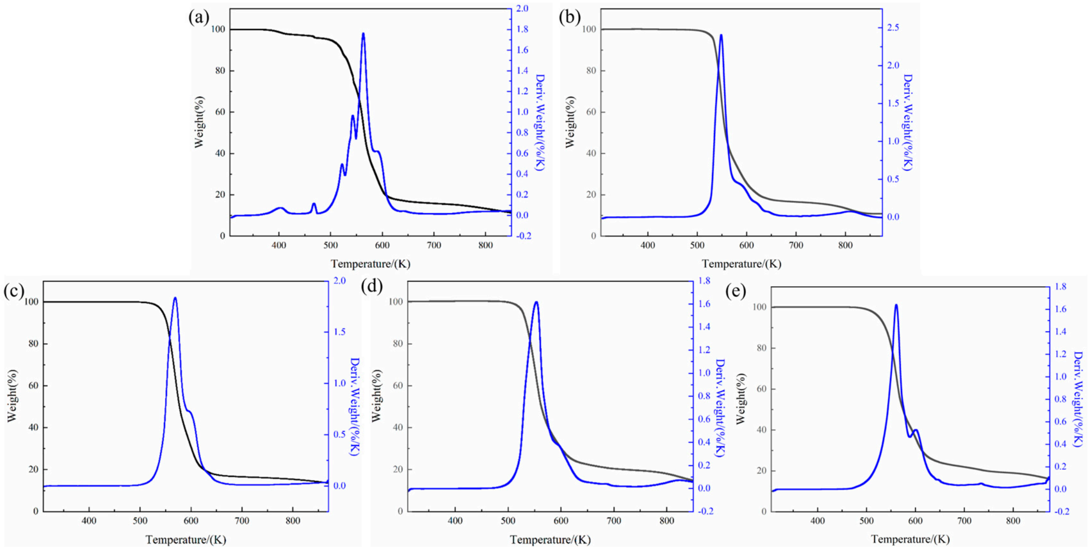

Figure S10 TG of compounds **1-5** (a) (b) (c) (d) (e)

Thermogravimetry (TG) is also known as the heat reduction method. In the temperature range of 300-800 K, when the heating rate is set to 10 K / min, the right amount of dry samples are selected, and the compounds **1-5** are subjected to thermogravimetric analysis test. The measured data are

plotted to obtain the TG and DTA curves shown in Figure 7 and Figure S9-S10. The analysis shows that the decomposition of compound **1** can be roughly divided into four stages. The first stage is in the range of 381-472 K, the weight loss rate is 4.23 %, which is basically consistent with the theoretical weight loss rate of 4.80 % of a molecule of acetonitrile. The second stage is in the range of 472-582 K, the weight loss rate is 62.02 %, which is basically consistent with the theoretical weight loss rate of 61.83 % of two molecules of crown ether. The third stage is in the range of 582-641 K, the weight loss rate is 16.14 %, which is basically consistent with the theoretical weight loss rate of 17.14 % of one molecule of ethylenediamine and two molecules of thiocyanate ions. In the fourth stage, in the range of 641-847 K, the weight loss rate is 5.75 %, which is basically consistent with the theoretical weight loss ratio of 4.92 % of a molecule of thiocyanate ion. After 847 K, it is the fifth stage, which is speculated that a molecule of thiocyanate ion and a molecule of manganese metal are not completely decomposed. The decomposition of compound **2** can be divided into four stages. In the first stage, the weight loss rate is 63.06 % in the range of 508-574 K, which is basically consistent with the theoretical weight loss rate of 63.85 % for two crown ethers. In the second stage, the weight loss rate is 20.28 % in the range of 574-670 K, which is basically consistent with the theoretical weight loss rate of 19.35 % for one molecule of propylene diamine and two molecules of thiocyanate ions. In the third stage, the weight loss rate is 5.25 % in the range of 670-831 K, which is basically consistent with the theoretical weight loss rate of 5.08 % for one molecule of thiocyanate ions. After 831 K, it is the fourth stage. It is speculated that a molecule of thiocyanate ion and a molecule of manganese metal are not completely decomposed ; The decomposition of compound 3 is roughly divided into three stages. In the first stage, the weight loss rate is 61.53 % in the range of 511-590 K, which is basically consistent with the theoretical weight loss rate of two molecular crown ethers of 62.87 %. In the second stage, the weight loss rate is 21.34 % in the range of 590-661 K, which is basically consistent with the theoretical weight loss rate of one molecule of propylene diamine and two molecules of thiocyanide ions of 20.68 %. After 661 K, it is the third stage. It is speculated that two molecules of thiocyanide ions and one molecule of metal manganese are not completely decomposed. The decomposition of compound **4** can be divided into four stages. In the first stage, the weight loss rate is 63.45 % in the range of 498-584 K, which is basically consistent with the theoretical weight loss rate of 61.75 % for two crown ethers. In the second stage, the weight loss rate is 16.37 % in the range of 584-697 K, which is basically consistent with the

theoretical weight loss rate of 17.07 % for one molecule of propylene diamine and one molecule of thiocyanate ion. In the third stage, the weight loss rate is 5.97 % in the range of 697-867 K, which is basically consistent with the theoretical weight loss rate of 4.91 % for one molecule of thiocyanate ion. After 867 K, it is the fourth stage. It is speculated that two molecules of thiocyanate ions and one molecule of manganese metal are not completely decomposed ; The decomposition of compound **5** can be divided into three stages. In the first stage, the weight loss rate is 58.14 % in the range of 491-588 K, which is basically consistent with the theoretical weight loss rate of 60.76 % for two crown ethers. In the second stage, the weight loss rate is 17.11 % in the range of 588-645 K, which is basically consistent with the theoretical weight loss rate of 18.31 % for one hexamethylenediamine and one thiocyanide ion. In the third stage, the weight loss rate is 8.77 % in the range of 645-873 K, which is basically consistent with the theoretical weight loss rate of 9.66 % for two thiocyanide ions. After 645 K, it is the third stage. It is speculated that a molecule of thiocyanate ion and a molecule of manganese metal are not completely decomposed ; thermogravimetric test results show that compounds **1-5** have good thermal stability.

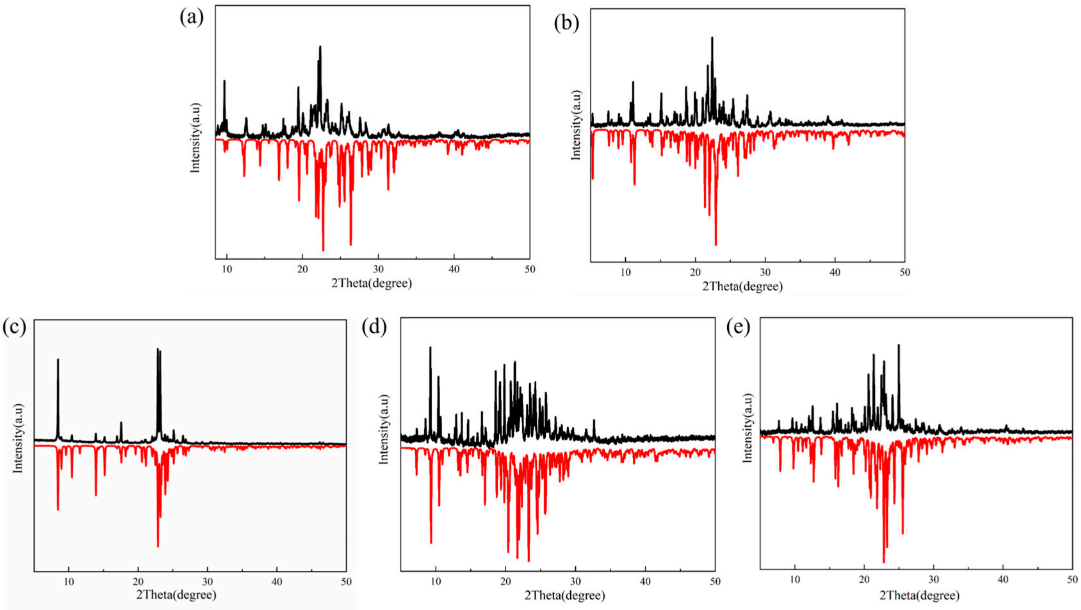

Figure S11 XRD patterns of compounds **1-5** (a) (b) (c) (d) (e)

As shown in Figure S11, the dry, pure and transparent samples of compounds **1-5** were selected for detection by X-ray single crystal diffractometer. The experimental analysis data of the obtained samples were compared with the simulated values deduced from the simulated single crystal structure. The degree of fitting is good, the relative intensity of the peak is also mostly consistent with the calculated peak position, and there are obvious characteristic peaks in the sample. The data

obtained from the analysis of the experimental results can further confirm that the compounds **1-5** samples are single pure phases.

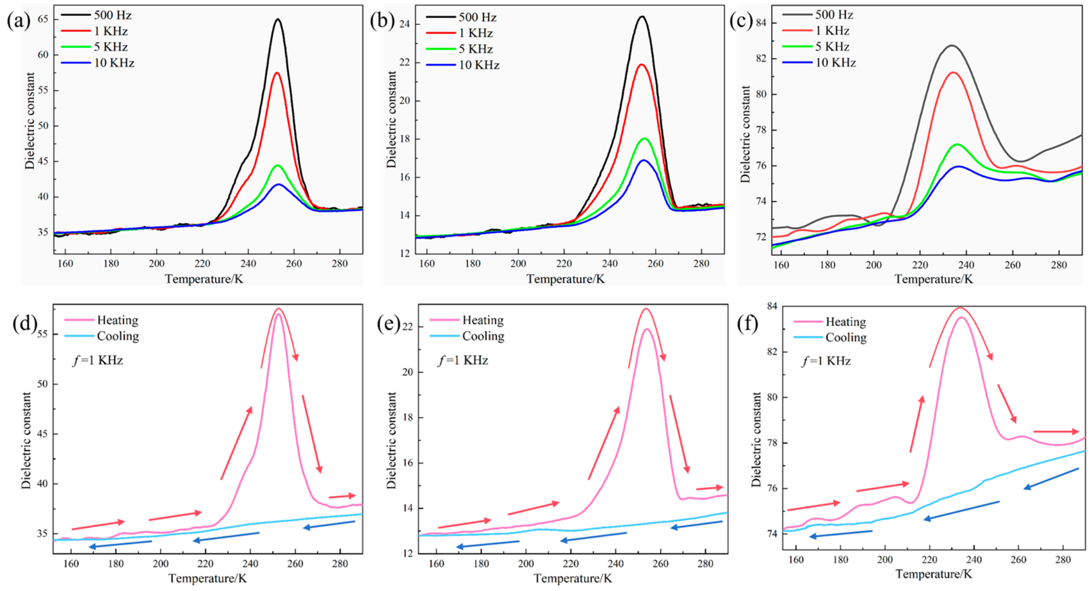

Figure S12 The dielectric constant curves of compound **1** in the a, b and c axis directions respectively (a) (b) (c)

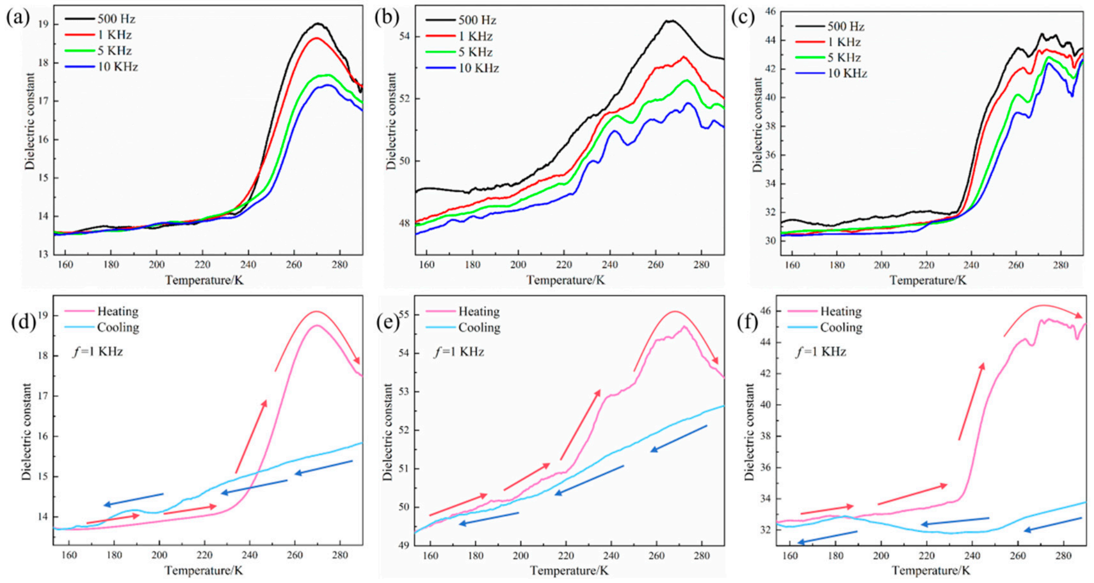

Figure S13 The dielectric constant curves of compound **2** in the a, b and c axis directions respectively (a) (b) (c)

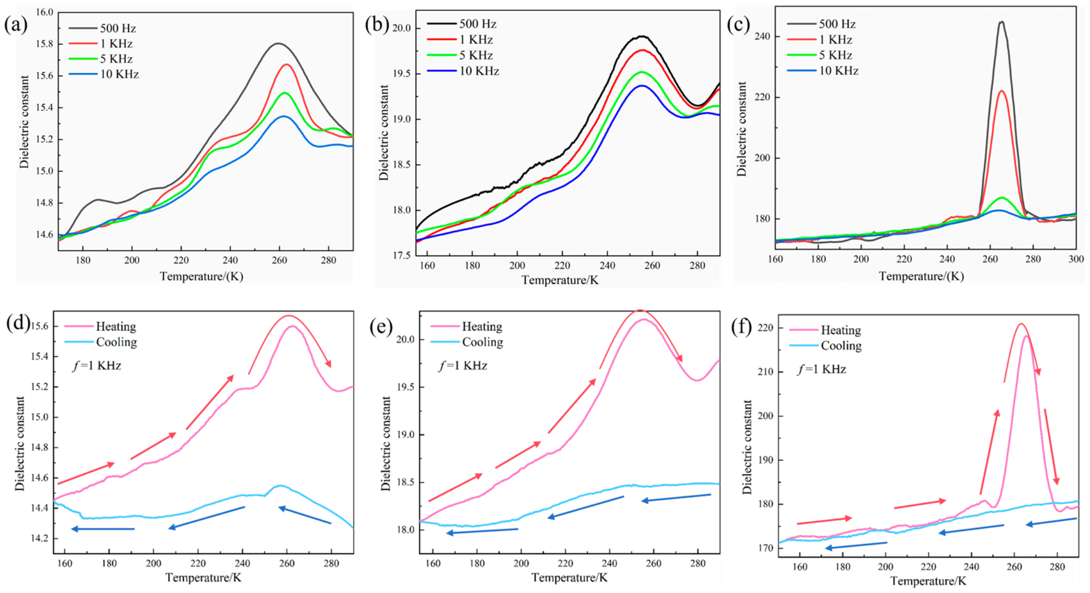

Figure S14 The dielectric constant curves of compound **3** in the a, b and c axis directions respectively (a) (b) (c)

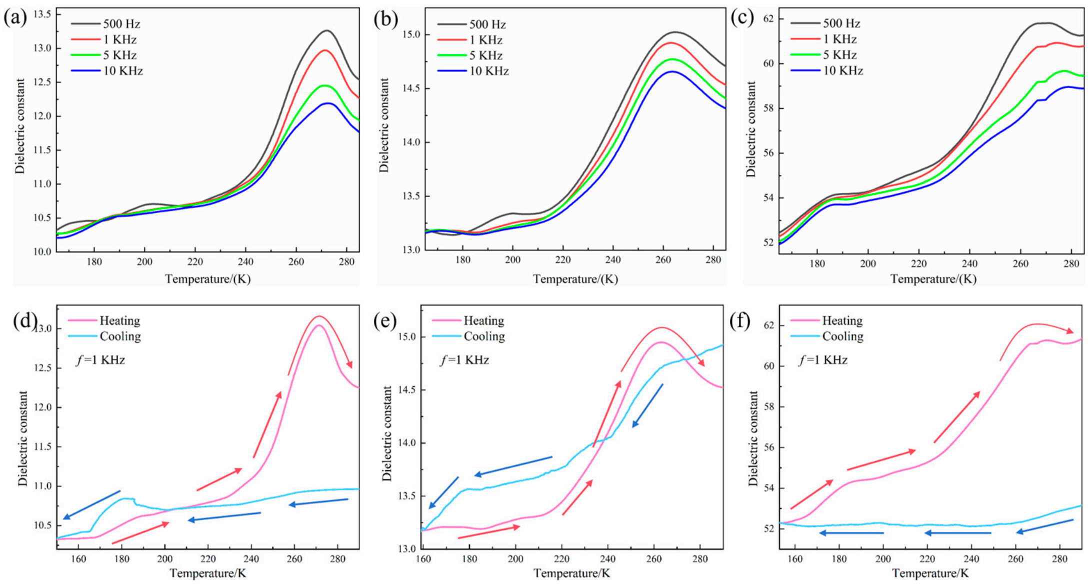

Figure S15 The dielectric constant curves of compound **4** in the a, b and c axis directions respectively (a) (b) (c)

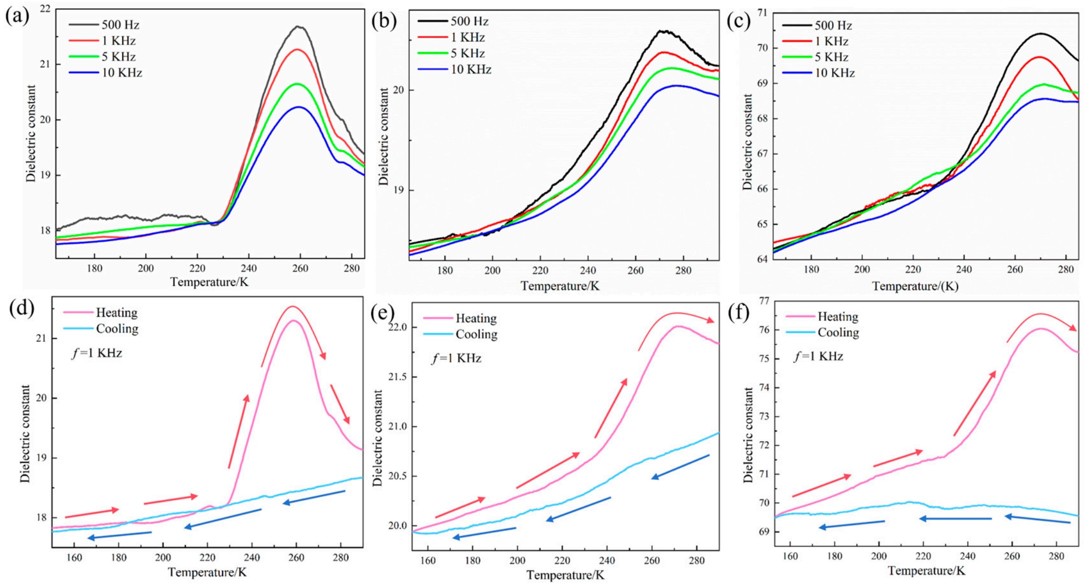

Figure S16 The dielectric constant curves of compound **5** in the a, b and c axis directions respectively (a) (b) (c)

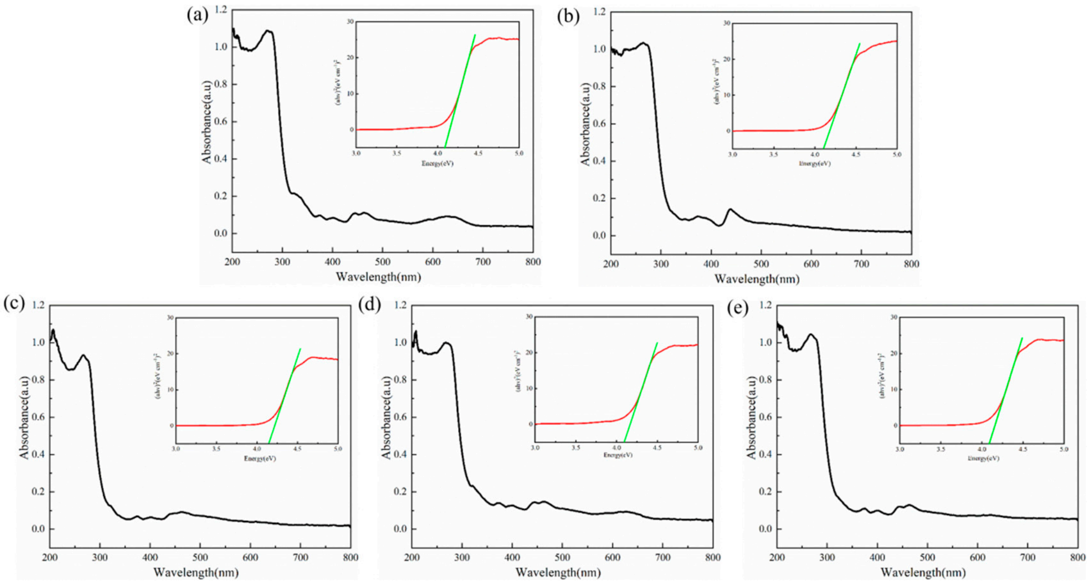

Figure S17 The solid UV spectra of compounds **1-5** (a) (b) (c) (d) (e)

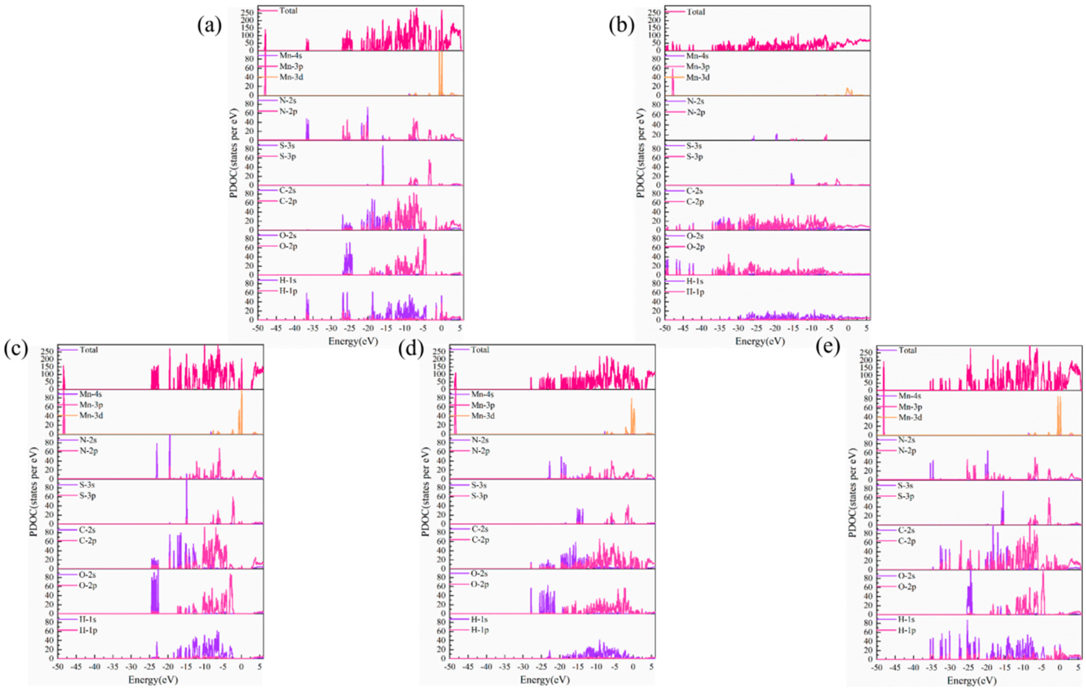

Figure S18 The density of states of compounds **1-5** (a) (b) (c) (d) (e)

Table S13 Compounds **1-5** Magnetic data

	C/cm^3 mol	Θ /K	μ_{eff}/μ_B
1	4.3863	7.1862	5.9237
2	4.4759	-12.0849	5.9839
3	4.2799	7.0116	5.8514
4	4.3952	3.0217	5.9297
5	4.2305	7.2197	5.8176

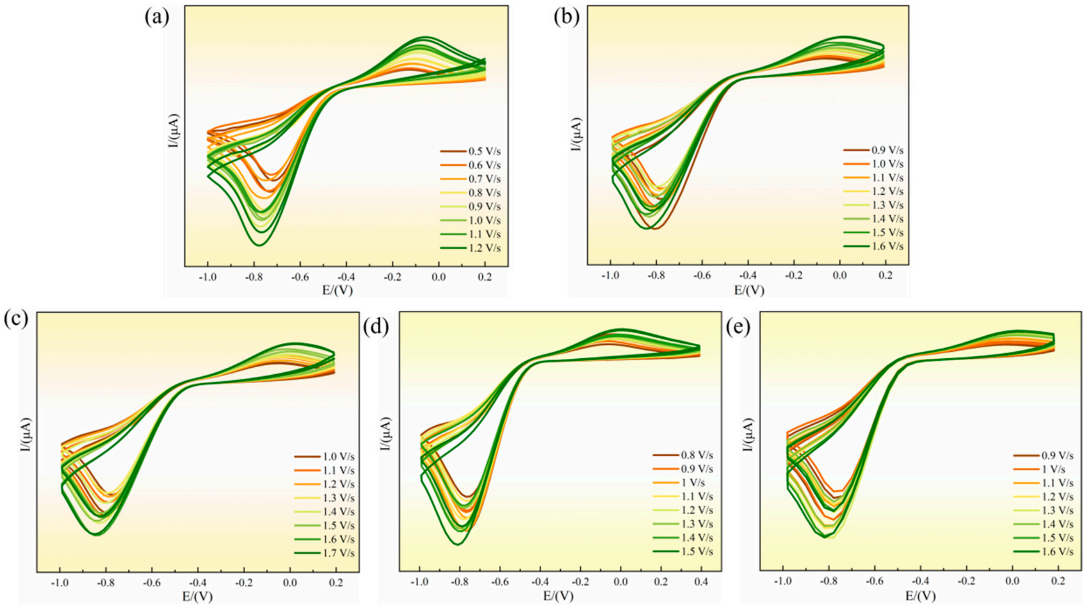

Figure S19 Cyclic voltammograms of compounds **1-5** (a) (b) (c) (d) (e)

In order to study the electrochemical properties of compounds **1-5**, a three-electrode system (glassy carbon electrode, auxiliary electrode, reference electrode) was used to test in a mixed solution of H_2SO_4 and Na_2SO_4. The cyclic voltammetry curves of compounds **1-5** are shown in Fig.S19. The test results show that there is a pair of obvious redox peaks in the potential range of-1.0 ~ 0.4 V. At the start of the scan, there is only a small amount of charging current. As the potential decreases, the reduction reaction occurs on the electrode surface, and the concentration of the reactant Mn^{2+} continues to decrease, while the concentration of the product Mn continues to increase until the cathode peak potential Epc is reached. At this time, the reduction rate on the electrode surface reaches the maximum, that is, the concentration polarization occurs completely, and then the potential continues to decrease. The current direction is reversed. With the positive shift of the applied potential, the reduced Mn on the electrode surface is reoxidized to Mn^{2+}, thus forming the anodic peak potential Epa.

When the scan rate is 1.0 V / s, the half-peak potential $E_{1/2}$ of compound **1** is-2.7646 mV ($E_{1/2}$= (Epa + Epc) / 2, | ΔEp | = Epa-Epc = 10.5036 mV. When the scan rate is 1.1 V / s, the half-peak potential $E_{1/2}$ of compound **1** is-2.5111 mV, | ΔEp | = 10.3317 mV. When the scan rate is 1.2 V / s, the half-peak potential $E_{1/2}$ of compound **1** is-3.303 mV, | ΔEp | = 12.7766 mV. At room temperature, $\Delta Ep=2.3\frac{RT}{nF}$ =6.4197 mV, $|\Delta Ep|>2.3\frac{RT}{nF}$, and it increases with the increase of v, and | ipa | $\neq$ | ipc |. Therefore, compound **1** is a quasi-reversible system.

When the scan rate is 1.0 V / s, the half-peak potential $E_{1/2}$ of compound **2** is-5.8181 mV, | ΔEp | = 15.9444 mV, when the scan rate is 1.1 V / s, the half-peak potential $E_{1/2}$ of compound **2** is-5.5847 mV, | ΔEp | = 16.8782 mV, when the scan rate is 1.2 V / s, the half-peak potential $E_{1/2}$ of compound **2** is-5.7973 mV, | ΔEp | = 17.2055 mV, at room temperature, $\Delta Ep=2.3\frac{RT}{nF}$ =6.6140 mV, $|\Delta Ep|>2.3\frac{RT}{nF}$, and it increases with the increase of v, and | ipa | $\neq$ | ipc |. Therefore, compound **2** is a quasi-reversible system.

When the scan rate is 1.0 V / s, the half-peak potential $E_{1/2}$ of compound **3** is-5.8065 mV, | ΔEp | = 15.8588 mV, when the scan rate is 1.1 V / s, the half-peak potential $E_{1/2}$ of compound **3** is-5.5692 mV, | ΔEp | = 16.9093 mV, when the scan rate is 1.2 V / s, the half-peak potential $E_{1/2}$ of compound **3** is-5.7094 mV, | ΔEp | = 17.1813 mV, at room temperature, $\Delta Ep=2.3\frac{RT}{nF}$ =6.5118 mV, |ΔEp|>2.3

$\frac{RT}{nF}$, and it increases with the increase of v, and | ipa | ≠ | ipc |. Therefore, compound **3** is a quasi-reversible system.

When the scan rate is 1.0 V / s, the half-peak potential $E_{1/2}$ of compound **4** is-9.1472 mV, | ΔEp | = 25.0575 mV, when the scan rate is 1.1 V / s, the half-peak potential $E_{1/2}$ of compound **4** is-9.3931 mV, | ΔEp | = 25.1127 mV, when the scan rate is 1.2 V / s, the half-peak potential $E_{1/2}$ of compound **4** is-9.9770 mV, | ΔEp | = 26.646 mV, at room temperature, ΔEp=2.3 $\frac{RT}{nF}$ =6.5118 mV, |ΔEp|>2.3 $\frac{RT}{nF}$, and it increases with the increase of v, and | ipa | ≠ | ipc |. Therefore, compound **4** is a quasi-reversible system.

When the scan rate is 1.0 V / s, the half-peak potential $E_{1/2}$ of compound **5** is-7.6606 mV, | ΔEp | = 18.9092 mV, when the scan rate is 1.1 V / s, the half-peak potential $E_{1/2}$ of compound **5** is-7.7618 mV, | ΔEp | = 19.8741 mV, when the scan rate is 1.2 V / s, the half-peak potential $E_{1/2}$ of compound **5** is-7.7095 mV, | ΔEp | = 20.4611 mV, at room temperature, ΔEp=2.3 $\frac{RT}{nF}$ =6.3160 mV, |ΔEp|>2.3 $\frac{RT}{nF}$, and it increases with the increase of v, and | ipa | ≠ | ipc |. Therefore, compound **5** is a quasi-reversible system. The above test results show that compounds **1-5** have good cycle stability and electrochemical properties.

Table S14 Electrochemical data of compounds **1-5**

		Epa	Epc	ΔEp	$E_{1/2}$
	1	2.4872	-8.0164	10.5	-2.8
1	1.1	2.6548	-7.6769	10.3	-2.5
	1.2	3.0850	-9.6916	12.8	-3.3
	1	2.1541	-13.7903	15.9	-5.8
2	1.1	2.8544	-14.0238	16.9	-5.6
	1.2	2.8055	-14.4000	17.2	-5.8
	1	2.1229	-13.7359	15.9	-5.8
3	1.1	2.8855	-14.0238	16.9	-5.6
	1.2	2.8813	-14.3000	17.2	-5.7
	1	3.3816	-21.6759	25.1	-9.1
4	1.1	3.1633	-21.9494	25.1	-9.4
	1.2	3.3460	-23.3000	26.6	-10.0
	1	1.7940	-17.1152	18.9	-7.7
5	1.1	2.1753	-17.6988	19.9	-7.8
	1.2	2.5211	-17.9400	20.5	-7.7
